# From immune desert to hot tumor: the tripartite synergy of tumor microenvironment-exosomes-immunogenic cell death in pioneering solutions

**DOI:** 10.3389/fimmu.2026.1738966

**Published:** 2026-04-07

**Authors:** Weitao Liu, Wenbo Xu, Wanglin Liu, Ruihan Liang, Shihan Tang, Xiangru Luo, Chuwei Liu, Qianhui Liao, Han Sun, Zhuoyan Wang, Chang Liu, Shuangjuan Liu, Huaqing Zhou, Yuqi Zhao, Guoming Zhang, Ming Yin, Huiping Liu

**Affiliations:** Key Laboratory of Oncology of Traditional Chinese Medicine, Hunan University of Chinese Medicine, Changsha, Hunan, China

**Keywords:** combination immunotherapy, exosomes, immune escape, immunogenic cell death (ICD), tumor microenvironment (TME)

## Abstract

The global incidence of cancer remains persistently high, with associated mortality rates remaining elevated owing to the challenges of early diagnosis and propensity for metastasis. The immunosuppressive “cold tumor” within the tumor microenvironment (TME), characterized by hypoxia, metabolic abnormalities, and immunosuppressive cellular infiltration, represents a key factor in treatment resistance and the failure of immunotherapies. Existing therapeutic approaches exhibit significant limitations that hinder curative outcomes. Tumor-derived exosomes (TEXs) frequently carry pro-cancer biomolecules, rendering single-exosome targeting strategies insufficient to reverse TME-mediated immunosuppression. Concurrently, danger signaling molecules released during immunogenic cell death (ICD) are readily neutralized by the immunosuppressive TME, resulting in inadequate and transient anti-tumor immune responses. Recent studies indicate that the TME, exosomes, and ICD do not function as isolated entities but rather constitute an interlinked signaling network. The TME modulates exosome biogenesis and release through hypoxic and inflammatory microenvironments while simultaneously attenuating the effects of ICD, thereby promoting immune evasion. Exosomes play a dual role in intercellular communication: TEXs amplify immunosuppressive signals, whereas engineered exosomes can deliver ICD inducers or immunomodulatory factors to reshape the immune state of the TME. ICD attempts to reverse TME suppression by releasing damage-associated molecular patterns (DAMPs); however, its effects require exosome-mediated long-range signal amplification and matrix penetration. Co-targeting the TME-exosome-ICD axis provides a mechanistic framework for enhancing the immunotherapy response by boosting DAMPs presentation, promoting antigen release, and facilitating immune cell infiltration. This approach also establishes a novel paradigm for reversing immunologically “cold” tumors towards an immunologically activated phenotype.

## 1 Introduction

The global burden of cancer continues to increase, posing major challenges for cancer treatment. According to statistics, cancer was responsible for 14.57% of all deaths worldwide in 2021, with lung, colorectal, gastric, liver, and breast cancers being the leading causes of cancer-related mortality (1). Countries such as China and the United States are experiencing particularly severe rises in cancer incidence and mortality, generating an urgent need for effective healthcare resource allocation and prevention strategies (2, 3). Although conventional chemotherapy can effectively kill tumor cells, it paradoxically remodels the tumor microenvironment (TME) and activates immunosuppressive mechanisms, ultimately compromising its therapeutic efficacy (4). Despite progress in immunotherapy, many tumors remain unresponsive. These often display an “immune desert” or “immune exclusion” phenotype, wherein immune cells fail to infiltrate the tumor efficiently or function adequately within it. For example, some patients develop resistance to PARP inhibitors or EGFR-targeted therapies through various molecular mechanisms, such as activation of alternative repair pathways and signaling bypasses (5, 6). In non-small cell lung cancer (NSCLC), resistance to immunotherapy has been associated with an increase in immunosuppressive cells and the formation of physical barriers in the TME (7). Key factors contributing to immunotherapy failure include the immunosuppressive nature of the TME, low tumor immunogenicity, and exosome-mediated signaling. The TME constitutes a complex ecosystem comprising diverse cellular and non-cellular components that collectively influence tumor initiation, progression, and treatment response (8, 9). As key mediators of intercellular communication, exosomes can transport molecules such as proteins and RNAs, thereby promoting tumor growth and immune evasion (10, 11).

In contrast, immunogenic cell death (ICD) is a form of cell death that elicits immune activation. It is characterized by extensive exposure, active secretion, or passive release of damage-associated molecular patterns (DAMPs), which can enhance anti-tumor immunity (12).

Recent studies have highlighted a close interplay between cancer stem cells (CSCs) and the TME. CSCs modulate the TME through various pathways, including exosome-mediated communication, promoting abnormal angiogenesis and immune suppression, thereby sustaining tumor drug resistance and recurrence potential (13).

Current therapeutic strategies struggle to fully disrupt the negative feedback loops between CSCs and their microenvironment, leading to limited clinical efficacy (14). Therefore, elucidating the synergistic mechanisms linking the TME, exosomes, and ICD, and developing combination interventions targeting key nodal points in these processes, could help convert “cold” tumors into “hot” tumors. Such an approach may enhance immune cell infiltration and improve treatment response, offering novel avenues to overcome tumor immune resistance.

## 2 Framework for the interaction of TME and ICD

The tumor microenvironment (TME) is a complex ecosystem composed of many cell types, extracellular matrix (ECM), and signaling molecules that has a significant regulatory impact on the efficacy and activation of immunogenic cell death (ICD). ICD effectiveness is impeded by metabolic abnormalities in the TME, such as oxidative stress and hypoxia, as well as a large number of immunosuppressive cells (15). This section first delineates how TME metabolic features govern ICD through key metabolites and cellular processes, followed by an examination of how matrix components and immunosuppressive cells shape the ICD response. (As illustrated in **Table 1**).

**Table 1:** Interaction framework between TME and ICD.

Interaction framework between TME and ICD	Primary regulatory aspects	Key factors/mechanisms
TME Regulation of ICD	Metabolites Induced by Hypoxia Control ICD	HIFs, Adenosine, Lactate, etc.
	Oxidative Stress and Metabolic Reprogramming Affect ICD	Oxidative Stress (ROS), Amino Acid Metabolism, Lipid Metabolism
	Immunosuppressive cells regulate ICD	Tregs, M2 Macrophages, MDSCs
	ICD Regulation Using TME Matrix Elements	Tumor Vasculature, Non-cellular Stromal Components, and Stromal Cells
	Single-Cell Insights	Discovery of EX-ICD Co-Expressing Subpopulations in the TME
ICD-mediated TME Remodeling	ICD-Released DAMPs Activate the TME Immune Response	DAMPs: CRT, HMGB1, ATP, etc.
	ICD-mediated TME Remodeling	DC Maturation and T-cell Infiltration, Macrophage polarization, NK Cell Activation
Application of the Framework	Clinical Translation Challenges of ICD Inducers	Dose Window and Safety Considerations
	Controversies and Reflections	Limitations of ICD Theory and Insights from Negative Results
	Clinical Significance and Future Perspectives	

### 2.1 TME regulation of ICD

Based on the “barrier-signal-cell” triad in tumor immunology, TME-mediated regulation of ICD operates through four interconnected dimensions. The four primary components of TME regulation of ICD are metabolic byproducts (such as hypoxia-induced adenosine and lactate), oxidative stress and metabolic reprogramming, immunosuppressive cell populations (such as Tregs, MDSCs, and TAMs), and physical matrix barriers (such as aberrant vasculature and dense extracellular matrix). These four layers constitute a “multi-layered defense system” against ICD, intricately linked through sophisticated communication networks. Hypoxia, for instance, simultaneously directly suppresses effector T cells via metabolic byproducts, recruits immunosuppressive cells through chemokine signaling, and promotes CAF-mediated ECM remodeling to establish physical barriers (16). Thus, developing successful combination therapy approaches necessitates an understanding of the intrinsic interactions between these four regulatory aspects.

#### 2.1.1 Metabolites induced by hypoxia control ICD

Hypoxia, a hallmark of TME, is primarily caused by abnormal vascular architecture and impaired perfusion as a result of rapid tumor growth (17). The hypoxia in the immunological microenvironment is aggravated by uncontrolled tumor angiogenesis, which disrupts the oxygenation balance (18).

Tumor hypoxia drives accumulation of HIFs, particularly HIF-1α, which orchestrate critical pro-tumorigenic programs.HIF-1α (19) is overexpressed in most human cancers and plays a vital role in hypoxia-induced signaling and tumor metastasis. Mutations in HIF-1α and p53 inhibit MDM2-mediated degradation, increasing HIF-1α stability and promoting tumor invasion, proliferation, and metastasis.

Hypoxia weakens the efficacy of ICD by activating the VEGF and CD39/CD73-adenosine axis via HIF-1α. This prevents DC maturation and CD8^+^ T cell infiltration, while increasing the immunosuppressive capacities of MDSCs and Tregs (19). Under hypoxic conditions, CD39/CD73 ectoenzymes generate abundant adenosine, which engages A2AR signaling to suppress T cells, NK cells, and DCs while activating MDSCs and Tregs, thereby promoting tumor cell survival, proliferation, and metastatic dissemination (19–21). Targeting the adenosine pathway with A2AR inhibitors or CD73 blockers can improve ICD-related immune responses by lowering MDSCs, enhancing CD8^+^ T cell infiltration, promoting DC activation, and reducing adenosine-mediated metabolic repression (19, 22). This TME-modifying strategy synergistically enhances both immune checkpoint inhibitors and photothermal therapy (23, 24).

Lactic acid accumulation, another key immunometabolic component of the TME, is driven by a glycolytic increase triggered by hypoxia (25). The Warburg effect accelerates tumor cell glycolysis, resulting in substantial amounts of lactic acid, which acidifies the TME and suppresses immune cell activation. Lactic acid, for example, enhances Treg inhibitory activity and increases PD-1 expression, allowing for immune evasion (26, 27). To improve ICD-induced T cell invasion and antitumor immunity, strategies that neutralize lactate or limit its transporters (such as lactate oxidase or MCT4 inhibitors) can alter the TME (28).

#### 2.1.2 Oxidative stress and metabolic reprogramming affect ICD

Beyond hypoxia-driven metabolite accumulation, the TME is characterized by pervasive oxidative stress and extensive metabolic reprogramming, both of which profoundly influence cell death pathways.

Oxidative stress regulates immune cell function and the progression of ICD by increasing the production of ROS. Different immune cells have distinct antioxidant systems and ROS sensitivity (29, 30). ROS exert dual functions in tumorigenesis: they stimulate T and NK cells, enhance ROS production, and recruit neutrophils and macrophages to eliminate cancer cells—thereby exerting antitumor effects (31); conversely, elevated ROS potentiate the immunosuppressive activity of MDSCs, TAMs, and Tregs, facilitating tumor immune evasion (32).

Many ROS-related tumor therapeutic techniques can be developed using the oxidative stress principles discussed above. For example, 2DG inhibits protein N-glycosylation, causing endoplasmic reticulum stress (ERS), which boosts photothermal treatment (PTT)-induced programmed ICD and improves immunotherapy efficacy (33). At the same time, tumor growth can be enhanced by leveraging ROS imbalance. When ROS oxidize and deactivate negative phosphatase regulators or directly oxidize kinases, they can activate the PI3K/AKT/mTOR and mitogen-activated protein kinase (MAPK) pathways, both of which are essential regulators of cell viability (34, 35).

Oxidative stress is closely linked to metabolic reprogramming of tumor cells, which includes lipid and amino acid metabolism in addition to glycolysis. Lipids can alter immune cells’ metabolic pathways, including CD8^+^ T lymphocytes, leading to immunosuppressive effects (36, 37). In contrast, lipids can have an indirect effect on immune activity by influencing other cells. Long-chain fatty acids delivered by tumor-derived extracellular vesicles via CD36 are absorbed by tumor-associated macrophages (MAMs), providing them with energy and commencing their pro-tumor activity (38). The enzymes IDO1 and TDO2, which are extensively expressed in the TME, eat tryptophan and generate Kyn (39, 40).

Kyn interacts with the AhR transcription factor via ligands on dendritic cells(DCs) and regulatory T cells (Tregs), causing AhR and HIF-1α to compete for the AhR nuclear translocation protein ARNT (41). This induces T-cell exhaustion. Furthermore, through AhR- and FoxP3-dependent mechanisms, kynurenine promotes differentiation of naive CD4^+^ T cells into immunosuppressive Tregs (42, 43). Quinolinic acid and 3-hydroxyanthranilic acid induce selective apoptosis in murine Th1 cells. Tryptophan depletion and Kyn accumulation promote Treg differentiation, suppress NK and effector T cells, and increase tolerance-inducing DC differentiation, all of which lead to tumor immunological tolerance (44, 45).

Despite the support of the IDO1/TDO-kyn mechanism (39, 40), other evidence suggests that TAM is activated by the arginine-polyamine metabolic axis via epigenetic reprogramming (for example, spermine regulates DNA demethylases). It is currently unclear how polyamines target specific gene loci in this approach and get through the TAM nuclear membrane (45).

#### 2.1.3 Immunosuppressive cells regulate ICD

Multiple immunosuppressive cell types enter the TME, establishing a barrier to ICD. This section will focus on three well-studied cell groups to investigate their regulatory capabilities within the TME and their impact on the ICD process: TAMs, MDSCs, and Tregs (15).

##### 2.1.3.1 Tregs

Tregs are a subset of CD4 T cells with a high inhibitory activity. They primarily suppress the immune system by releasing cytokines such as TGF-β, IL-10, and IL-35 (46, 47). TGF-β inhibits tumor growth and spread by lowering the cytotoxicity of NK cells and cytotoxic T lymphocytes (CTLs) and encouraging NK cell conversion to type I innate lymphoid cells inside the TME (46, 47). Tregs that generate IL-10 suppress cytotoxic effector activities of effector T cells, but Tregs that release IL-35 promote effector T cell exhaustion (48, 49).

Tregs block antitumor immune responses, accelerating tumor development (50). Tregs are enriched in the TME through two mechanisms: first, chemokines attract them to the tumor site, and second, the TME’s unique metabolic environment (e.g., acidity and metabolite depletion) promotes Treg proliferation, allowing them to outnumber effector CD4^+^ T cells (51). This feature allows Tregs to continue suppressing even in the acidic and metabolically deficient TME (52).

Targeted pharmacological manipulation of Tregs is becoming a key strategy in tumor immunotherapy (53, 54). Imatinib inhibits the phosphorylation of lymphocyte-specific protein tyrosine kinase (LCK) and reduces the number of Tregs, decreasing TCR signaling in these cells (55).

##### 2.1.3.2 MDSCs

MDSCs are an important type of immunosuppressive cell in the TME, where they promote tumor immune evasion and disease development through a variety of mechanisms. Inflammatory mediators such as PGE2, G-CSF, and TGF-β attract immature myeloid cells (IMCs) and block their differentiation into mature myeloid cells, resulting in a distinct population of MDSCs within the TME (56). Inflammatory signals stimulate IKKβ, which phosphorylates and degrades ARID1A, a chromatin remodeling factor. This process promotes MDSC chemotaxis and aggregation by blocking the NF-κB pathway, silencing negative regulators including A20, and releasing CXCR2 ligands such as CXCL1 and CXCL2 (57).

MDSCs carry out a number of tasks. Castration-resistant prostate cancer (CRPC) is accelerated by the interleukin-23 (IL-23) they produce, which protects tumor cells from aging-related growth arrest (58, 59). Monocytic MDSCs migrate to tumor tissues and differentiate into TAMs in response to microenvironmental stresses such as hypoxia. Local cytokines—including LPS, TNF-α, and IFN-γ—influence TAM polarization toward pro-inflammatory M1 or immunosuppressive M2 phenotypes, thereby modulating ICD progression (60). DAMPs generated during ICD, such as following radiation therapy, have been shown to upregulate the expression of programmed death-ligand 1 (PD-L1) on MDSCs, thereby increasing their immunosuppressive effect (61), revealing a negative feedback loop between ICD and adaptive immune suppression.

##### 2.1.3.3 M2 macrophages

TAMs suppress dendritic cell maturation by producing anti-inflammatory cytokines such as IL-10 and TGF-β1, as well as chemokines such as CCL17, CCL18, and CCL22. This lowers antigen presentation ability and increases Treg recruitment, hence strengthening tumor immune suppression (62, 63).

The primary pro-tumor feature of M2-type TAMs is polarization, which is regulated by many signaling pathways. Lactate promotes the mTORC2 and ERK signaling pathways in the hypoxic TME, promoting TAM polarization to the M2 phenotype. Activated M2-type TAMs release CCL17 via the CCL17/CCR4/mTORC1 axis, which promotes pituitary adenoma invasion (64, 65). Furthermore, exosomes can promote M2 polarization. When MDSCs and mouse bone marrow cells are co-cultured with exosomes from lung adenocarcinoma (LLC) cells, they grow into M2-type macrophages, according to studies (66). In colorectal cancer (CRC), NAMPT stabilizes HIF-1α, causing M2-type TAMs to polarize.

M2-type TAMs promote tumor growth by producing a number of effector chemicals such as matrix metalloproteinases like MMP-2 and MMP-9, as well as proangiogenic factors like vascular endothelial growth factor and epidermal growth factor. These substances work together to inhibit T and NK cell function while also increasing angiogenesis and tumor progression (67–69). Furthermore, M2-type TAM-derived heme oxygenase-1 (HO-1) and cyclooxygenase-2 (COX-2) exhibit pro-tumor activity. As a result, focusing on TAM polarization has become an important strategy for improving anti-tumor immunity. For example, R848 can reprogram M2-type TAMs to an M1 phenotype, increasing their phagocytic capacity against oxaliplatin-treated lung cancer cells (70).

#### 2.1.4 ICD regulation using TME matrix elements

TME, which comprises both tumor vasculature and matrix components, exerts a substantial impact on the induction and efficacy of ICD (71, 72).

Tumor vasculature frequently exhibits disorganized architecture and overt leakage (71). These blood vessels preferentially recruit immunosuppressive cells, including MDSCs, TAMs, and Tregs, while also exacerbating tissue hypoxia (73). Within the TME, these cells collaborate to suppress anticancer immune responses, thereby promoting immune escape and tumor progression (71, 74).

Matrix cells and acellular matrix components within the TME work together to form biochemical and physical barriers that regulate ICD. The most prevalent matrix cells in the TME are cancer-associated fibroblasts (CAFs), which exert multiple regulatory effects on ICD. First, CAFs physically impede the infiltration of ICD effector cells. Tumor cells secrete growth factors to recruit normal fibroblasts into the TME, which then differentiate into CAFs that promote matrix rigidity and ECM deposition (75). CAFs form dense matrix networks by secreting and remodeling the ECM, including the deposition of large amounts of collagen and hyaluronic acid (HA). This not only inhibits the penetration and distribution of ICD inducers (e.g., chemotherapeutic agents and nanomedicines) into tumor tissue but also directly blocks the infiltration of CTLs into tumor nests (75–77). In addition to acting as a physical barrier to drug penetration, the stiffer matrix compresses microvessels, further limiting drug distribution; reducing the deposition of ECM components such as HA can improve chemotherapy efficacy (76, 77). Second, CAFs directly attenuate ICD-induced immune responses by secreting immunomodulatory molecules.

CAFs secrete transforming growth factor-β (TGF-β), which enhances tumor cell invasion, inhibits the mTOR pathway, suppresses the development and function of natural killer (NK) cells, and sustains their inactivation (78–80).

In addition to CAFs, the ECM plays a crucial role in regulating ICD. Composed of collagen, HA, and other substances, the ECM not only forms a physical barrier but also directly modulates immune cell function via its chemical components. Current research indicates that collagen acts as both a physical structure and an immunosuppressive signal, directly affecting T cell activity. When excessively accumulated type I collagen in tumor tissues binds to the inhibitory receptor leukocyte-associated immunoglobulin-like receptor 1 (LAIR-1) on the surface of immune cells, it inhibits the antitumor immune response induced by ICD, limiting T cell proliferation and effector function. Furthermore, heat shock protein 47 (HSP47), which is highly expressed by tumor cells, promotes collagen deposition in the TME by facilitating proper collagen folding and secretion.

The α2β1 integrin/NF-κB pathway promotes the polarization of immunosuppressive microglia/macrophages, which indirectly suppresses CD8^+^ T cell activation. To reverse collagen-mediated immunosuppression and enhance ICD efficacy, strategies targeting collagen synthesis (e.g., reducing HSP47 expression) or blocking collagen-immune cell interactions (e.g., using the LAIR-2 fusion protein NC410) have shown promise (81, 82).

Regarding HA, intact HA at high concentrations may exert immunosuppressive effects, whereas degraded HA fragments may be immunostimulatory. Excessive HA exacerbates hypoxia, impairs immune cell infiltration, increases interstitial pressure, and compresses blood vessels (76). Thus, targeting ECM remodeling—for instance, using hyaluronidases (e.g., PEGPH20) to degrade HA or pirfenidone (PFD) to inhibit CAF activity and ECM deposition—has emerged as an important adjuvant strategy to overcome physical barriers, improve drug delivery, and enhance the efficacy of ICD-based immunotherapies (76, 83).

#### 2.1.5 Single-cell insights: discovery of EX-ICD co-expressing subpopulations in the TME

Previous discussions on TME heterogeneity have largely been confined to bulk RNA sequencing data and traditional histopathological analysis. While these methods can reveal overall gene expression patterns and cell type proportions, they obscure the finer molecular heterogeneity among individual cells (84). Specifically, bulk sequencing data fail to distinguish gene co-expression patterns within specific cell subpopulations, resulting in a macro-level understanding of TME complexity that struggles to precisely identify key effector cells driving immune responses or immune escape (85).

In recent years, the rapid advancement of single-cell transcriptomics (scRNA-seq) technology has provided unprecedented resolution for deciphering TME heterogeneity (85). By integrating recent scRNA-seq studies on tumors such as hepatocellular carcinoma (HCC) and non-small cell lung cancer (NSCLC), we can deeply explore the co-expression patterns of ICD-related genes and exosomal markers within specific cell subpopulations, thereby uncovering previously unrecognized functional units within the TME (86).

Recent scRNA-seq studies have identified a distinct tumor cell subpopulation within the TME of HCC and NSCLC. These cells simultaneously overexpress ICD-associated genes (e.g., CRT, HMGB1) and exosome markers (e.g., RAB27A, CD63) (87). In HCC studies, single-cell analysis identified a tumor cell subset with high expression of CRT and HMGB1 that also highly expressed RAB27A. These cells effectively activated DCs and promoted CD8^+^ T cell infiltration by secreting exosomes enriched with tumor antigens and DAMPs (88). In NSCLC, similar studies identified specific tumor cell subpopulations co-expressing HMGB1 and CD63. These cells not only readily undergo ICD themselves but also transmit immunosuppressive molecules such as miR-21 via exosomes, thereby impairing immune cell function within the TME (16).

Additionally, this phenomenon can also be observed in other tumors. For example, in pancreatic ductal adenocarcinoma, scRNA-seq analysis identified a tumor cell subpopulation that simultaneously overexpresses the ICD marker HMGB1 and the key exosome secretion gene RAB27A. These cells release HMGB1-rich exosomes that not only directly activate DCs but also promote M2 polarization of TAMs via exosome-mediated delivery of miR-21-5p, thereby establishing an immunosuppressive microenvironment (89). In CRC, scRNA-seq studies identified a tumor cell subset co-expressing CRT and CD63. These cells trigger DC maturation by exposing CRT while secreting CD63^+^ exosomes carrying PD-L1, thereby suppressing effector T cell function and establishing a dual regulatory network of “immune activation-suppression” (90).

Specifically, RAB27A, as a key regulator of exosome biogenesis and secretion, plays a central role in exosome release across multiple tumor cell lineages (91). CD63, a member of the tetramembrane protein family, serves as a classic surface marker for exosomes, participating in their sorting and targeted delivery (92). CRT, a key marker of ICD, exhibits high expression closely associated with tumor cell exposure of the “eat me” signal, promoting phagocytosis of dying tumor cells by DCs (93). HMGB1 activates immune cells upon extracellular release, enhancing antitumor immune responses (94).

This co-expression pattern of EX-ICD suggests these cells may function both as “initiators” of ICD (releasing DAMPs via ICD) and as “messengers” in intercellular communication (transmitting bioactive molecules via exosomes), thereby exerting dual regulatory roles within the TME (95). Single-cell evidence further indicates that this EX-ICD co-expressing subpopulation significantly correlates with T cell exhaustion scores, immunosuppressive microenvironment formation, and poor patient prognosis. This suggests they may remodel immune cell function locally or distantly via exosome-mediated DAMPs transfer (96).

**Figure 1:**
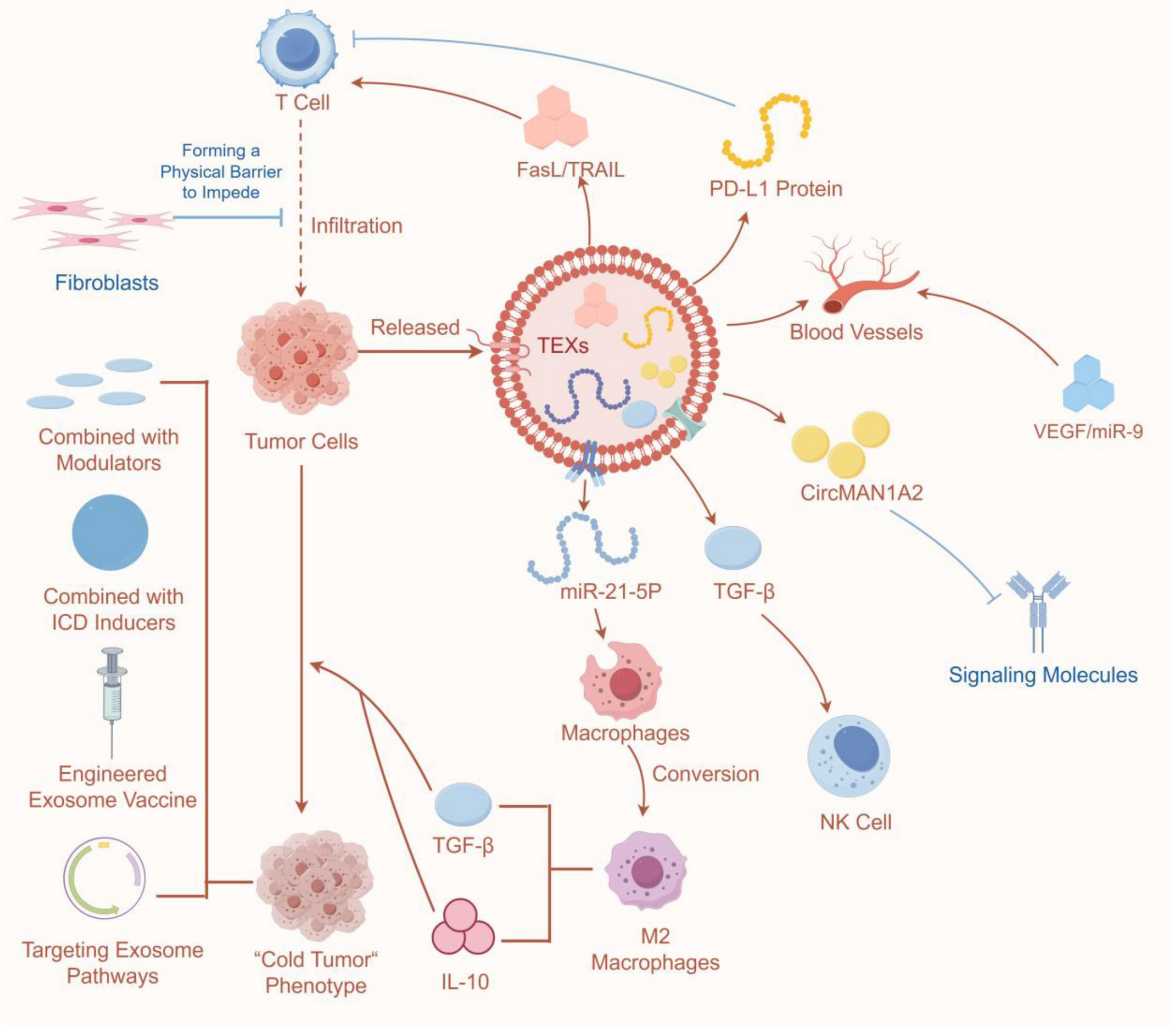
Schematic overview of TME-mediated regulation of ICD.

The discovery of this EX-ICD co-expressing subpopulation provides cellular-level evidence for understanding TME heterogeneity. These cells represent not only a convergence point for immune responses and immune evasion within the TME but also potential key targets for combination therapeutic strategies (97). Targeting this cell population could simultaneously block both their ICD-induced immune activation and exosome-mediated immunosuppression, thereby more effectively reshaping the TME and enhancing antitumor immunotherapy efficacy. This discovery establishes a robust cellular foundation for subsequent studies on the synergistic mechanisms of EX-ICDs and offers new perspectives for developing novel tumor immunotherapies (98). (As illustrated in **Figure 1**).

Reading Pathway: Tumor cells release TEXs, which establish an immunosuppressive microenvironment through multiple mechanisms, ultimately leading to the formation of “cold tumors”.

(This figure illustrates the mechanisms by which TEXs regulate the TME by inhibiting ICD, as well as corresponding potential intervention strategies. The left panel depicts how microenvironmental factors, including hypoxia, metabolic reprogramming, and oxidative stress, directly or indirectly suppress ICD induction. The central panel illustrates how immunosuppressive cell populations (Tregs, MDSCs, and M2-type TAMs) impair DC maturation and CTL function by secreting inhibitory cytokines or expressing immunosuppressive molecules. The right panel focuses on matrix components: CAFs form physical barriers via ECM deposition and secrete factors such as TGF-β, collectively shaping an immunosuppressive TME that leads to the “cold tumor” phenotype and markedly attenuates ICD efficacy. Furthermore, TEXs act as critical intercellular communicators, transmitting immunosuppressive signals throughout the regulatory network and further exacerbating ICD inhibition. Collectively, this figure systematically summarizes how TEX-mediated immunosuppression disrupts ICD, along with the core intervention strategy of targeting TEXs to restore ICD and reverse the “cold tumor” phenotype).

### 2.2 ICD-mediated TME remodeling

#### 2.2.1 ICD-released DAMPs activate the TME immune response

The release or exposure of several damage-associated molecular patterns (DAMPs) from dying cells is a key feature of ICD. PRRs on immune cells detect these molecules, initiating an adaptive immune response. Classic DAMPs include secreted ATP, high-mobility group box protein B1 (HMGB1) — which is released during late apoptosis — and calreticulin (CRT), which is exposed on the cell surface during the preapoptotic phase. Furthermore, heat shock proteins (HSPs), including HSP70 and HSP90, are essential for ICD. As molecular chaperones, their functions extend beyond simply assisting cells in surviving under harsh conditions. When HSP70/90 is secreted extracellularly or expressed on the cell surface, it can act as an effective immune adjuvant. They enhance T-cell responses by promoting antigen cross-presentation through binding to receptors on antigen-presenting cells (APCs), such as CD91 (99).

##### 2.2.1.1 CRT

Whether soluble or non-specifically internalized by living cells, the CRT protein cannot directly induce DC maturation or activation. To appropriately stimulate the response of APCs, its immunomodulatory function requires the co-release of additional DAMPs during the ICD process, resulting in an “immunogenic signaling combination” (100). The exposure of CRT on cancer cell surfaces is considered an important “eat me” signal in tumor immunotherapy, as it enhances DC phagocytosis of dying tumor cells. By binding to its receptor on APCs, this signal promotes antigen uptake and processing through the activation of endocytic signaling pathways (101).

During the ICD process, membrane-bound CRT supports the migration of antigen-loaded immature DCs to draining lymph nodes by interacting with scavenger receptors on the surface of APCs. This initiates a tumor-specific adaptive immune response by promoting antigen cross-presentation, activating naive CD8^+^ T cells, and guiding their maturation into CTLs (102, 103). However, soluble CRT may act as a decoy, competing with membrane-bound CRT for binding to APC receptors. This impairs the establishment of anticancer immunity by inhibiting the effective clearance of apoptotic cells and increasing the accumulation of immunosuppressive cells in the spleen and peripheral tissues (104).

A variety of therapeutic modalities, including radiotherapy (105), photodynamic therapy (PDT) (106), specific chemotherapeutic agents (e.g., doxorubicin, oxaliplatin) (107), and physical therapies such as nanomaterial-mediated photothermal therapy (33), have successfully induced CRT membrane translocation. Studies have shown that radiation can induce tumor cells to produce oxidized phospholipid-rich microparticles, leading to ferroptosis-like changes that promote ATP release and CRT membrane exposure. This enhances the phagocytosis of tumor cells by macrophages, providing new insights into the combination of immunotherapy and radiotherapy (105). Furthermore, although Bip/GRP78 inhibition combined with ionizing radiation enhances DC maturation and antigen presentation, it may also increase the release of ICD-associated DAMPs, such as CRT exposure, ATP secretion, and HMGB1 release (108). In particular, PDT uses ROS generated by photosensitizers to effectively induce endoplasmic reticulum stress and accelerate CRT exposure. It also synergistically upregulates molecules such as HSP60/70, resulting in robust antitumor immune responses (106, 109). *In vitro* studies using a 3D pancreatic cancer model have demonstrated that photodynamic immunotherapy with TR-PINs can activate antitumor responses in both CD4^+^ and CD8^+^ T cells by inducing the expression of CRT, HSP60, HSP70, and HMGB1 in a light-dose- and time-dependent manner (106).

##### 2.2.1.2 HMGB1

One of the most important DAMP molecules is the highly conserved nucleoprotein HMGB1 (110). It exerts a dual role in the TME: attracting and activating immunosuppressive cells to facilitate tumor immune escape, while also enhancing antitumor immunity by activating DCs and T cells.

By binding to its receptors (including TLR4 and RAGE), HMGB1 activates NK cells, promotes macrophage polarization toward the M1 phenotype, and suppresses M2 polarization. It also facilitates the maturation of DCs and enhances their antigen presentation capacity. Activated DCs further stimulate CD8^+^ T lymphocytes, enabling them to infiltrate tumors and exert cytotoxic effects. Activated T cells and NK cells secrete proinflammatory cytokines such as IFN-γ and TNF-α, which form positive feedback loops among immune cells and elicit robust antitumor immune responses.

Within tumor tissues, HMGB1 frequently exhibits abnormal cytoplasmic distribution, suggesting that it is actively secreted or released upon cell death. The extracellular form of HMGB1 can activate signaling pathways that regulate DC maturation and antigen presentation by binding to PRRs such as TLR4 and RAGE (111). Chemotherapy, radiotherapy, and PDT have all been shown to induce HMGB1 release from tumor cells, which is essential for maintaining the immunogenicity of ICD (112).

##### 2.2.1.3 ATP

Adenosine triphosphate (ATP) is a DAMP released during ICD, primarily modulating immune responses in the TME through the activation of purinergic receptors, including the P2X and P2Y families (113).

Extracellular ATP (eATP) in the TME serves as a critical “find-me” signal. By binding to specific receptors such as P2Y2R, it triggers signaling pathways involved in chemokine secretion and immune cell migration, thereby recruiting immune cells—including neutrophils, macrophages, and DCs—to accumulate at the tumor site (114, 115). In general, elevated eATP levels are associated with enhanced antitumor immunity (116). Studies have demonstrated that high concentrations of eATP can induce pyroptosis in TAMs expressing P2X7R. This process eliminates local immunosuppressive cells and consequently promotes T cell-mediated antitumor immune responses (117, 118).

ATP enhances immune activation through multiple mechanisms. As an immune adjuvant during ICD, eATP activates the NLRP3 inflammasome via the P2X7 receptor, thereby promoting the secretion of pro-inflammatory cytokines such as interleukin-1β (IL-1β). This, in turn, enhances the recruitment and maturation of DCs. When combined with immune checkpoint inhibitors, eATP can further promote T cell infiltration into the tumor parenchyma (118). In T cells, eATP binds to P2X7R on their surface, activating the MAPK and nuclear factor of activated T cells (NFAT) signaling pathways, which promotes T cell proliferation, cytokine secretion, and cytotoxic activity (119).

##### 2.2.1.4 HSP60

In addition to classic HSP70 and HSP90, HSP60—a member of the heat shock protein family primarily localized in mitochondria—also exerts a significant immunoregulatory role during ICD. Under stressful conditions, HSP60 can be released from dying cells or exposed on the cell surface, acting as DAMPs molecule to initiate immune responses (120). Studies have demonstrated that apoptotic cells induced by UV irradiation or cisplatin treatment rapidly upregulate membrane-bound HSP60. This membrane-associated HSP60 promotes the maturation of bone marrow-derived DCs (characterized by upregulated CD80 and CD86 expression) and stimulates the secretion of pro-inflammatory cytokines such as IL-6 and IL-1β (121).

In terms of functional mechanisms, membrane HSP60 binds to its co-receptor LOX-1 (lectin-like oxidized low-density lipoprotein receptor 1) to mediate dendritic cell uptake of apoptotic cells and promote cross-presentation of cellular antigens, thereby amplifying the CD8^+^ T cell response (121). Notably, the immunostimulatory activity of HSPs is tightly linked to their purity: highly purified HSPs lack direct cytokine-inducing capacity, with their effects partly deriving from associated immunogenic peptides or contaminants (122). Nevertheless, the early surface exposure of HSP60 on apoptotic cells (preceding phosphatidylserine externalization) makes it a key “eat-me” signal co-factor during ICD. Together with CRT, it acts on APCs to initiate antitumor immunity (121).

#### 2.2.2 ICD-driven dynamic changes of immune cells within the TME

##### 2.2.2.1 DC maturation and T-cell infiltration

ICD enhances antitumor immune responses through the synergy of multiple mechanisms, with DCs maturation and effective T cell infiltration as critical steps. DAMP signals released during ICD synergize with intracellular pattern recognition pathways: for example, Mn²^+^ generated by MnO_2_ degradation in the acidic TME acts as a cGAS co-activator, enhancing its binding affinity to cytosolic DNA. This promotes cGAS–STING pathway activation, increases type I interferon (IFN-I) production, and thereby drives DC maturation and antigen cross-presentation (123).

As a novel form of ICD, ferroptosis releases early signaling molecules (e.g., oxidized phospholipids) that promote the maturation and activation of bone marrow-derived DCs (BMDCs) via pathways such as TLR4/MyD88, enhancing antigen-specific T cell proliferation and cytotoxicity (124). Lipid peroxides (e.g., 4-HNE, MDA) from ferroptotic cells are taken up by DCs to modify antigenic peptides, boosting MHC-I-mediated antigen presentation, accelerating CD8^+^ T cell priming, and facilitating tumor control (125). Currently, nanomaterials, engineered biological carriers, and immunosuppressive microenvironment reshaping strategies provide novel approaches to promote DC maturation and enhance ICD-induced immunity (126–128). However, the threshold mechanisms governing bidirectional regulation of ferroptosis-induced ICD—such as lipid peroxidation extent and macrophage metabolic state—remain elusive (129, 130).

##### 2.2.2.2 Macrophage polarization

Macrophage polarization, particularly M2-to-M1 reprogramming, is a critical bridge linking various forms of cell death (e.g., ICD, pyroptosis, regulated necrosis) to antitumor immune responses (131).

As discussed in Section 1.1.3.3, M2-type TAMs in the TME promote immunosuppression via secretion of IL-10 and TGF-β. ICD disrupts this immunosuppressive state, driving M1 repolarization, with ICD-induced DAMPs serving as key signals. For example, HMGB1 binding to TLR4 facilitates M1 polarization while inhibiting M2 (110). Additionally, IL-1β, IL-18 and DAMPs derived from pyroptosis induce M1 conversion via activation of the NLRP3 inflammasome and NF-κB signaling, thereby transforming “cold” to “hot” tumors and enhancing lymphocyte infiltration (132).

Based on this mechanism, therapeutic strategies utilize ICD to drive M2-to-M1 repolarization. For example, the nano-inducer IMZF dissociates in the acidic, GSH-rich TME, releasing ions that alleviate hypoxia and generate ·OH via the Fenton reaction, thus depleting local GSH. These changes activate the HIF-1α/NF-κB pathway, promoting M1 polarization, DC maturation and T cell infiltration, and ultimately reversing immunosuppression (133). Similarly, the TLR7/8 agonist R848 directly reprograms M2 TAMs to the M1 phenotype, enhancing their phagocytic capacity against oxaliplatin-treated lung cancer cells (70).

##### 2.2.2.3 NK cell activation

Macrophage polarization, particularly M2-to-M1 reprogramming, is a critical bridge linking various forms of cell death (e.g., ICD, pyroptosis, regulated necrosis) to antitumor immune responses (131).

As discussed in Section 1.1.3.3, M2-type TAMs in the TME promote immunosuppression via secretion of IL-10 and TGF-β. ICD disrupts this immunosuppressive state, driving M1 repolarization, with ICD-induced DAMPs serving as key signals. For example, HMGB1 binding to TLR4 facilitates M1 polarization while inhibiting M2 (110). Additionally, IL-1β, IL-18 and DAMPs derived from pyroptosis induce M1 conversion via activation of the NLRP3 inflammasome and NF-κB signaling, thereby transforming “cold” to “hot” tumors and enhancing lymphocyte infiltration (132).

Based on this mechanism, therapeutic strategies utilize ICD to drive M2-to-M1 repolarization. For example, the nano-inducer IMZF dissociates in the acidic, GSH-rich TME, releasing ions that alleviate hypoxia and generate ·OH via the Fenton reaction, thus depleting local GSH. These changes activate the HIF-1α/NF-κB pathway, promoting M1 polarization, DC maturation and T cell infiltration, and ultimately reversing immunosuppression (133). Similarly, the TLR7/8 agonist R848 directly reprograms M2 TAMs to the M1 phenotype, enhancing their phagocytic capacity against oxaliplatin-treated lung cancer cells (70).

NK cells, as key effector cells of innate immunity, possess the ability to rapidly recognize and eliminate aberrant cells without the need for prior sensitization, playing a pivotal role in ICD-induced antitumor immunity. Their activation is not only dependent on the engagement of activating receptors (such as NKG2D and NCRs) with stress-induced ligands (e.g., MICA/B and ULBP) on the surface of target cells but is also finely regulated by the interaction between inhibitory receptors (such as KIRs and CD94/NKG2A) and MHC class I molecules. This delicate balance enables NK cells to effectively discriminate between normal and malignant cells, thereby preventing autoimmune tissue damage (134, 135). Activated NK cells secrete substantial amounts of cytokines, including IFN-γ and TNF-α, which modulate the microenvironment via signaling pathways such as JAK-STAT, thereby promoting dendritic cell maturation and T cell function while enhancing adaptive immune responses (136).

Type I interferons (IFN-I) serve as important regulators of NK cell function. By activating STAT1/STAT4 signaling pathways through the IFNAR receptor, they upregulate the expression of perforin and granzyme B in NK cells, thereby enhancing their cytotoxic activity against tumor cells (137).

**Figure 2:**
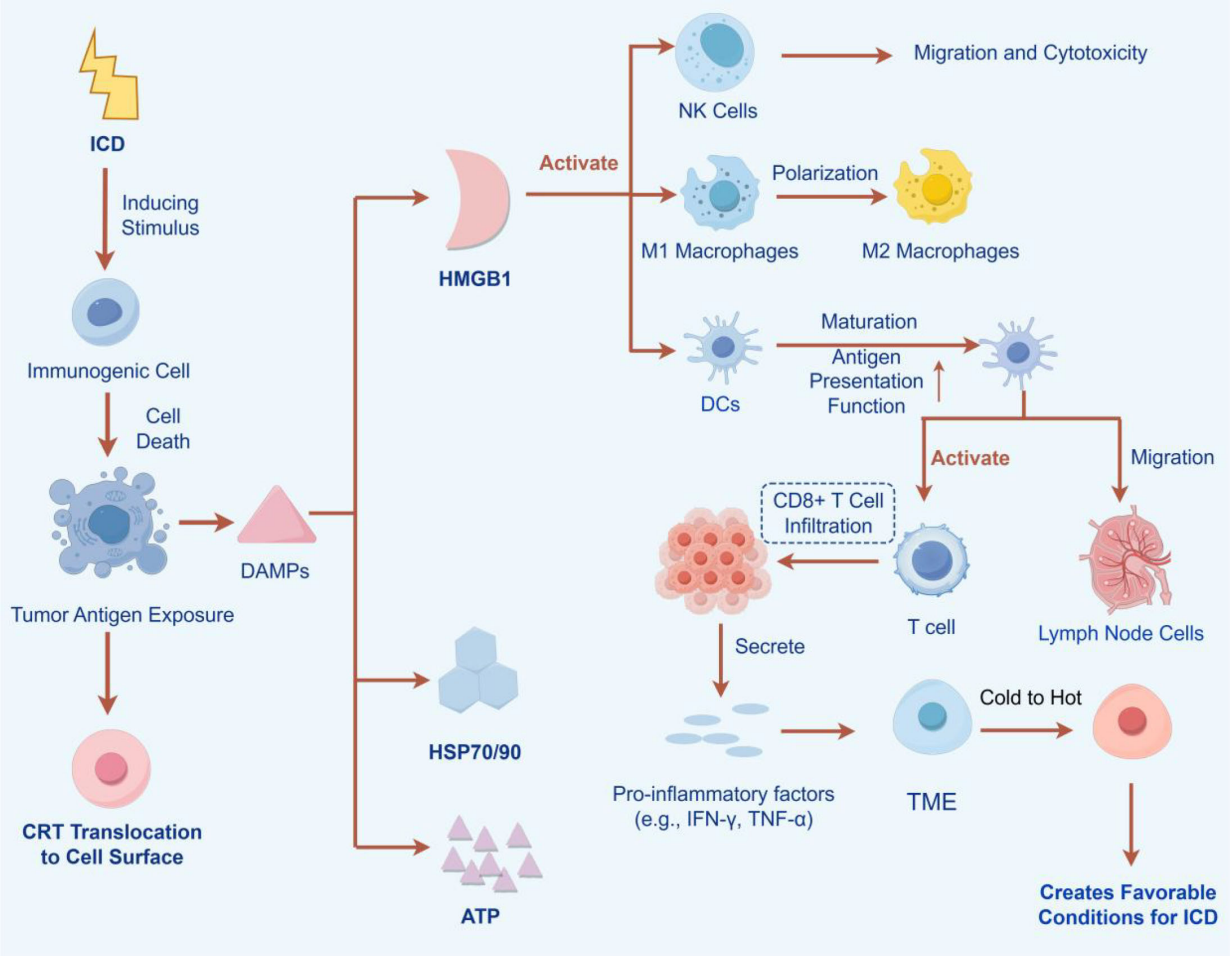
Schematic diagram illustrating the mechanism by which ICD remodels the TME.

Extracellular vesicles, such as exosomes, exert dual roles in the regulation of NK cells. Tumor-derived exosomes carrying molecules like HSP70 can promote IFN-γ production in NK cells via the TLR2-NF-κB pathway, enhancing their migration and cytotoxicity (138). Conversely, certain TDEs may suppress NK cell activity and promote immune evasion by delivering TGF-β or expressing NKG2D ligand homologs (139). (As illustrated in **Figure 2**).

Reading Pathway: ICD induces the release of tumor antigens and DAMPs, activating immune cells, which in turn promote CD8^+^ T cell infiltration into tumors, thereby converting “cold tumors” into “hot tumors”.

(This figure illustrates the mechanism by which ICD inducers trigger immunogenic cell death to activate antitumor immunity and remodel the tumor microenvironment. This process is accompanied by the release and exposure of a series of DAMPs: ① “Eat-me” signals: CRT and HSP70/90 are exposed on the cell surface, promoting DC-mediated phagocytosis of tumor antigens; ② “Find-me” signals: ATP is released into the extracellular space, recruiting immune cells such as DCs to the tumor site; ③ Immune activation signals: HMGB1 is released and binds to receptors such as TLR2/4. These DAMPs act synergistically to promote DC maturation and antigen cross-presentation. Mature DCs migrate to lymph nodes and activate tumor-specific CD8^+^ T cells. Activated CTLs and NK cells infiltrate the tumor tissue and secrete IFN-γ and TNF-α, which not only kill tumor cells but also further remodel the TME, driving macrophage polarization from the M2 to the M1 phenotype. Ultimately, this complete cascade transforms immunologically “cold” tumors (characterized by scarce immune cell infiltration) into “hot” tumors (enriched with effector immune cells and highly responsive to immunotherapy).

### 2.3 Application of the framework

#### 2.3.1 Clinical translation challenges of ICD inducers

In the context of ICD-based therapy, the profiles of damage-associated molecular patterns elicited by different ICD inducers may vary. For instance, photodynamic therapy has been demonstrated to effectively induce the expression of multiple DAMPs—including HSP60, HSP70, calreticulin, and high mobility group box 1 protein—thereby activating T cell-mediated antitumor immune responses (140). Similarly, various conventional chemotherapeutic agents, such as doxorubicin and oxaliplatin, exert their immunogenic effects by inducing CRT exposure, ATP and HMGB1 release, as well as stimulating type I interferon responses (141).

Although numerous ICD inducers have demonstrated potent antitumor immune activation potential in preclinical studies, their clinical translation still faces critical challenges. Central to these challenges is the therapeutic window between the “minimum ICD dose” and the “maximum tolerated dose.” For example, the capacity of classic chemotherapeutic drugs like doxorubicin and oxaliplatin to induce ICD exhibits significant dose dependency: doses below the threshold may fail to effectively induce CRT exposure and ATP release, whereas doses approaching or exceeding the maximum tolerated dose, while potentially enhancing immunogenicity, can lead to severe adverse effects such as irreversible myelosuppression, cardiotoxicity, or neurotoxicity, ultimately compromising overall immune function (141).

Similarly, the ICD-inducing efficacy of photodynamic therapy is closely related to light dosage, photosensitizer concentration, and oxygenation levels: excessively low doses may only trigger non-immunogenic apoptosis, while excessively high doses can cause tissue necrosis and release DAMPs. However, the accompanying intense inflammatory response and tissue damage may disrupt established adaptive immune responses (142). Consequently, identifying the “immunologically optimal biological dose”—one that maximizes ICD effects while maintaining acceptable toxicity—has emerged as a research hotspot. This endeavor necessitates integrating dynamic biomarker monitoring and real-time immune surveillance to enable personalized dose adjustments (143).

#### 2.3.2 Limitations of ICD theory and insights from negative results

In the context of ICD-based therapy, the profiles of DAMPs elicited by different ICD inducers may vary. For instance, PDT has been demonstrated to effectively induce the expression of multiple DAMPs—including HSP60, HSP70, CRT, and HMGB1—thereby activating T cell-mediated antitumor immune responses (140). Similarly, various conventional chemotherapeutic agents, such as doxorubicin and oxaliplatin, exert their immunogenic effects by inducing CRT exposure, ATP and HMGB1 release, as well as stimulating type I interferon responses (141).

Although numerous ICD inducers have demonstrated potent antitumor immune activation potential in preclinical studies, their clinical translation still faces critical challenges. Central to these challenges is the therapeutic window between the “minimum ICD dose” and the “maximum tolerated dose.” For example, the capacity of classic chemotherapeutic drugs like doxorubicin and oxaliplatin to induce ICD exhibits significant dose dependency: doses below the threshold may fail to effectively induce CRT exposure and ATP release, whereas doses approaching or exceeding the maximum tolerated dose, while potentially enhancing immunogenicity, can lead to severe adverse effects such as irreversible myelosuppression, cardiotoxicity, or neurotoxicity, ultimately compromising overall immune function (141).

Similarly, the ICD-inducing efficacy of PDT is closely related to light dosage, photosensitizer concentration, and oxygenation levels: excessively low doses may only trigger non-immunogenic apoptosis, while excessively high doses can cause tissue necrosis and release DAMPs. However, the accompanying intense inflammatory response and tissue damage may disrupt established adaptive immune responses (142). Consequently, identifying the “immunologically optimal biological dose”—one that maximizes ICD effects while maintaining acceptable toxicity—has emerged as a research hotspot. This endeavor necessitates integrating dynamic biomarker monitoring (e.g., CRT-positive exosomes and HMGB1 levels in peripheral blood) and real-time immune surveillance to enable personalized dose adjustments (143).

Although the ICD theory provides an important framework for cancer immunotherapy, its clinical translation still faces numerous controversies and challenges, with some studies reporting negative results that contradict the classical paradigm. First, not all instances of cell death involving CRT exposure are immunogenic. For example, studies have shown that certain apoptosis inducers, while capable of triggering CRT translocation to the cell membrane, fail to coordinately induce the release of ATP and HMGB1, and thus cannot effectively initiate DC maturation and T cell responses (144, 145).

Second, the efficacy of ICD inducers exhibits significant heterogeneity across tumor types and among individuals. In multiple myeloma research, even when tumor cells were treated with established ICD inducers to prepare vaccines, complete protection was not achieved in tumor-bearing mice; only delayed tumor progression was observed. This suggests that within a highly immunosuppressive TME, ICD induction alone may be insufficient to overcome intrinsic immune tolerance (146). More notably, certain therapeutic modalities may simultaneously induce both ICD and immunosuppressive effects, resulting in a “double-edged sword” phenomenon. For instance, while radiotherapy induces ICD in tumor cells, it can also upregulate PD-L1 expression on tumor cells via activation of the NF-κB pathway and recruit MDSCs into the TME, thereby diminishing its immune-activating effects (147).

Similarly, certain chemotherapeutic agents at specific doses, while capable of inducing ICD, may also significantly suppress bone marrow hematopoietic precursors, leading to lymphopenia and ultimately counteracting any immune-enhancing effects (148). These contradictory negative findings indicate that ICD induction is merely the initial step in antitumor immune responses; the ultimate outcome depends on the dynamic balance between immune-activating and immunosuppressive signals within the TME. Future research should shift from a linear “single ICD induction” perspective to a systematic “ICD-TME multidimensional interaction” framework, focusing on how combination therapeutic strategies can amplify ICD effects by remodeling the TME while circumventing potential immunosuppressive side effects (149).

#### 2.3.3 Clinical significance and future perspectives

In summary, a bidirectional, dynamic, and highly complex interactive network exists between the TME and ICD. The TME restricts the initiation and efficacy of ICD through multiple mechanisms, including hypoxic metabolites, oxidative stress, immunosuppressive cells, and physical barriers. Conversely, ICD reshapes the TME by releasing DAMPs, driving the polarization of immune cells toward antitumor phenotypes. A deeper understanding of the molecular mechanisms underlying this interactive network not only helps explain the limited clinical efficacy of single ICD inducers but also provides a theoretical foundation for developing novel combination therapeutic strategies. Future research should focus on: 1. developing biomarkers for dynamic monitoring of TME-ICD interactions to enable patient stratification and efficacy prediction; 2. investigating sequential combination regimens of ICD inducers with TME-targeting agents to disrupt immunosuppressive barriers; and 3) validating ICD-TME interaction mechanisms in clinically relevant complex models to bridge the gap between preclinical research and clinical translation (16, 150). (As illustrated in **Table 2**).

**Table 2:** Drugs and intervention strategies regulating TME-ICD interactions.

Strategies/agents	Mechanism of action	Research model	Evidence of ICD	Immune effects	References
A2AR antagonist	Blocks adenosine binding to A2AR, thereby relieving immune cell suppression	Mouse tumor model	Promotes DC activation	Increased intratumoral CD8^+^ T cell infiltration, decreased MDSCs, and enhanced ICD-mediated antitumor immunity	(19)
CD73 inhibitor (e.g., AMPCP)	Inhibits CD73 enzymatic activity, reducing adenosine production	Mouse model of colon cancer	Synergizes with PTT to induce ICD	Alleviates functional impairment of DCs, synergistically stimulates T cell activation, and inhibits distant tumor growth	(23)
Lactate oxidase (LOX@ZIF-8@MPN)	Catalyzes lactate degradation and converts the byproduct H_2_O_2_ to •OH	Mouse model of breast cancer	•OH generation triggers ICD	Normalizes tumor vasculature; increases T cell infiltration	(359)
Zn-UCMB	Selectively binds and consumes lactate; promotes singlet oxygen generation	Mouse model of breast cancer	Enhances PDT-induced ICD	Downregulates HIF-1α and VEGF expression; promotes TAM re polarization from M2 to M1 phenotype	(360)
MG-LAAO	Neutralizes H^+^ and inhibits lactate accumulation, alleviating TME acidosis	Mouse model of melanoma	Activates the cGAS-STING pathway	Remodels immunosuppressive TME; prevents immune evasion	(28)
MCT4 inhibitor (tropane alkaloids)	Downregulates MCT4 expression, blocking lactate efflux	Mouse model of lung cancer	Enhances ferroptosis via intracellular acidosis, indirectly promoting ICD	Alleviates immunosuppression; enhances T lymphocyte infiltration stimulated by ICD	(361)
CuSe/CoSe nanomaterial (releasing Syrosingopine)	Depletes GSH; decomposes H_2_O_2_ to •OH triggering ferroptosis; simultaneously blocks lactate efflux	Mouse model of breast cancer	Enhances ferroptosis via intracellular acidosis, indirectly promoting ICD	Alleviates immunosuppression; enhances ICD-stimulated T lymphocyte infiltration	(361)
IDO1 inhibitor (e.g., epacadostat)	Inhibits IDO1 activity, reducing tryptophan-to- kynurenine metabolism	Multiple tumor models (preclinical and clinical)	Restores effector T cell function	Reduced kynurenine production; enhanced CAR-T cell function; alleviated tumor immunosuppression	(39, 362)
TDO2 inhibitor	Inhibits TDO2 activity, blocking the tryptophan-kynurenine metabolic axis	Mouse model of esophageal squamous cell carcinoma	Reduces M2 macrophage polarization	Inhibits AKT/GSK3β pathway activation; downregulates IL-8 expression; slows tumor progression	(40)
2-Deoxyglucose (2DG)	Inhibits glycolysis and protein N-glycosylation, inducing ER stress	Mouse model of breast cancer	Synergistically enhances PTT-induced ICD	Reverses tumor thermotolerance; improves immunotherapy efficacy	(33)
Imatinib	Inhibits LCK phosphorylation, disrupting TCR signaling	Mouse tumor model	Reduces Treg numbers	Impairs Treg suppressive function	(55)
Low-dose cyclophosphamide	Selectively depletes Tregs	Multiple tumor models (clinical)	—	Reduces circulating Tregs; increases tumor-specific T cell ratio	(53)
GDF15 blocker (targeting CD48)	Blocks GDF15 binding to CD48 on Tregs	Mouse model of liver cancer	—	Attenuates Treg function; alleviates liver cancer-associated immunosuppression	(54)
R848	TLR7/8 agonist	Mouse model of lung cancer	—	Reprograms M2-type TAMs toward the M1 phenotype; enhances phagocytosis of oxaliplatin-treated tumor cells	(70)
Bevacizumab (anti-VEGF)	Neutralizes VEGF, promoting vascular normalization	Multiple tumor models (clinical)	Improves ICD efficacy in combination therapies	Improves vascular function; enhances response to anti-PD-1/PD-L1 therapy	(363, 364)
DC-101 (anti-VEGFR2)	Blocks VEGFR2 signaling; low-dose induces vascular normalization	Mouse models of breast cancer and glioma	—	Increases pericyte coverage; improves tumor perfusion; promotes T cell infiltration	(365)
Pirfenidone (PFD)	Inhibits TGF-β signaling; reprograms pancreatic stellate cells	Mouse model of pancreatic cancer	—	Reduces ECM deposition; enhances nanomedicine penetration; optimizes CTL-mediated antitumor immunity	(83)
NO delivery system	Releases nitric oxide; degrades tumor collagen	Mouse model of breast cancer	—	Facilitates uniform delivery of IDO inhibitor (NLG919); suppresses PTT-induced IDO upregulation	(366)
Hyperbaric oxygen (HBO)	Increases tumor oxygenation levels	Mouse tumor model	—	Promotes accumulation of engineered bacteria in tumors; enhances PTT and ICD-induced immunotherapy	(367)
LIFU-TMD	Disrupts tumor vasculature via low-intensity focused ultrasound	Mouse model of breast cancer	Induces CRT exposure in partial tumor cells	Depletes tumor blood supply; enhances anti-PD-L1 immunotherapy efficacy	(368)
MnO_2_ nanoparticles	Degrades in acidic TME to release Mn²^+^	Multiple mouse tumor models	Mn²^+^ activates the cGAS-STING pathway	Enhances type I interferon response; promotes DC activation and T cell priming	(369)
Oncolytic virus OH2-FLT3L	Intratumoral injection of FLT3L-expressing oncolytic virus	Mouse model of melanoma	Induces ICD in tumor cells	Releases tumor antigens; promotes DC antigen cross-presentation; enhances T cell activation	(370)
HK2-engineered T cells	Overexpresses hexokinase 2 (HK2), enhancing glycolysis	Mouse tumor model	—	Increases cytokine secretion; enhances activation marker expression; enhances metabolic activity; enhances antitumor efficacy	(371)
cdG/Mix nanoformulation	Delivers c-di-GMP, triggering pyroptosis in cancer cells	Mouse model of CRC	Induces ICD	Reverses T cell exhaustion; activates NK and CD8^+^ T cells	(128)
Zinc oxide nanocomposite	Selectively induces ICD in melanoma cells	Mouse model of melanoma	CRT exposure, HMGB1 release, ATP secretion	Promotes DC maturation; increases CD4^+^ and CD8^+^ T cell infiltration into tumors	(126)
Iron oxide nanocomposite (under AMF)	Heats to 45 °C under alternating magnetic field, inducing mild hyperthermia	Mouse tumor model	Triggers ICD	Activates CTLs and NK cells; inhibits Tregs; induces M1 macrophage polarization	(372)
HELA-Exo (engineered exosomes)	Engineered exosomes loaded with tumor antigens	Mouse tumor model	—	Activates cDC1s; cross-presents tumor antigens; induces tumor-reactive CD8^+^ T cell responses	(127)
ALDH1A1 inhibitor	Inhibits ALDH1A1 activity, blocking TAK1 phosphorylation	Mouse tumor model	—	Reduces GM-CSF secretion; inhibits MDSC expansion	(373)
NAMPT inhibitor	Inhibits NAMPT activity, reducing HIF-1α stabilization	Mouse model of CRC	—	Blocks M2-type TAM polarization; impairs immunosuppressive microenvironment	(374)
SDF-1/CXCR4 inhibitor (e.g., AMD3100)	Blocks SDF-1 secreted by CAFs from binding to CXCR4	Mouse model of pancreatic cancer	—	inhibits SATB-1 expression; reduces malignant progression and drug resistance in pancreatic cancer	(375)
Hyaluronidase (e.g., PEGPH20)	Degrades hyaluronan in the ECM	Mouse model of pancreatic cancer and clinical trials	—	Reduces matrix deposition; improves drug penetration; enhances ICD-induced antitumor immunity	(76)
Ferroptosis inducer (e.g., erastin)	Induces ferroptosis in tumor cells	Mouse tumor model	Releases lipid peroxides (e.g., 4-HNE, MDA)	Enhances DC uptake and processing of tumor antigens; accelerates CD8^+^ T cell activation	(124)
Oncolytic virus VSV	Selectively replicates in and lyses tumor cells	Mouse model of melanoma	CRT exposure, ATP and HMGB1 release	Combined with NKT cell activation; improves survival; reduces metastatic burden	(376)
HSP70-modified exosomes	Exosomes surface-displaying HSP70	*In vitro* and mouse models	—	Stimulates NK cell migration and cytotoxicity via the TLR2/NF-κB pathway	(99)
Q-DOX (doxorubicin nanoformulation)	Controlled-release doxorubicin	Mouse tumor model	CRT exposure, ATP and HMGB1 release	Activates DCs; induces tumor-specific immune responses	(107)
TLR2 inhibitor	Blocks HSP70-mediated TLR2 activation	*In vitro* study	—	Inhibits NK cell hyperactivation induced by multiple myeloma-derived vesicles	(138)
CX3CR1 inhibitor	Blocks CX3CL1-CX3CR1 interaction	Mouse model of ovarian cancer	—	Inhibits angiogenesis and macrophage recruitment; slows tumor progression	(377)
PLK1 inhibitor	Disrupts macrophage-naive CD4^+^ T cell interaction	Mouse tumor model	—	Inhibits differentiation of naive CD4^+^ T cells into Tregs; blocks immunosuppressive microenvironment formation	(378)

## 3 Framework of interaction between exosomes and TME

TME and exosomes interact in a bidirectional and dynamic way. Signals such as hypoxia and inflammation within the TME have a major impact on exosome synthesis and cargo sorting; nevertheless, exosomes, as key intercellular communication mediators, reciprocally alter the TME’s cellular composition and functional state. Understanding the interaction structure is required for determining the mechanisms that drive tumor growth and developing treatment approaches. This section will describe the regulatory mechanisms of TME on exosome secretion, the remodeling effects of exosomes on the TME, and the practical obstacles this interaction framework faces in clinical translation, all based on recent research findings. (As illustrated in **Table 3**).

**Table 3:** Interaction framework between TME and exosomes.

Interaction framework between TME and exosomes	Primary regulatory aspects	Key factors/mechanisms
TME regulates exosomal secretion	Regulation of microenvironmental signals	Hypoxia, inflammation
	Signaling Routes	Signaling pathway regulation STAT3 pathway, Ras/Raf/MEK/ERK pathway, Wnt/β-catenin pathway
Exosome-Mediated TME Remodeling	Tumor-promoting effect	Inhibition of T cell activity, induction of M2 macrophage polarization, and promotion of angiogenesis
	Tumor-suppressive function	Engineering exosomes for STAT6 ASO delivery, delivery of carbonyl manganese, and intelligent responsive drug delivery
Clinical Challenges of the TME-ICD Interplay	Clinical Translation of the TME-Exosome Axis	Liquid biopsy, therapeutic intervention, and treatment monitoring
	Theoretical Limitations	Limitations of 2D models, Exosome heterogeneity, Risks of non-selective inhibition, Functional duality, Unclear biogenesis mechanisms
	Future Perspectives	Multidimensional intervention strategies, closed-loop management throughout the entire disease course , Single-vesicle multi-omics technologies, Standardized manufacturing systems

### 3.1 Terminology definition

Extracellular vesicles (EVs) are a heterogeneous group of membrane-bound vesicles. Traditionally, they are categorized into exosomes (30–150 nm, derived from the endosomal pathway), microvesicles (100–1000 nm, originating from plasma membrane budding), and apoptotic bodies (>1 μm) based on their biogenesis processes (151, 152). However, existing isolation methods cannot fully purify a single subtype, and recent studies have revealed significant overlap in size, density, and protein markers among multiple EV subpopulations. For instance, numerous microvesicles express low levels of markers typically associated with exosomes, and their size distributions often overlap (153).

The International Society for Extracellular Vesicles (ISEV) published the MISEV 2023 guidelines, which recommend the use of operational terminology to avoid confusion arising from uncertain subcellular origins. The guidelines define “small EVs” (sEVs) as vesicles smaller than 200 nm isolated by density gradient centrifugation, size-exclusion chromatography (SEC), or differential ultracentrifugation, and “medium/large EVs” (m/lEVs) as vesicles larger than 200 nm. The term “exosome” can only be used when definitive endosomal markers are present or ESCRT-dependent biogenesis is demonstrated. Similarly, the term “microvesicle” should only be employed when their origin is confirmed to be plasma membrane budding (153).

Currently, the vast majority of tumor-related publications referring to “exosomes” or “tumor-derived exosomes (TDEs)” actually describe mixed populations of small EVs isolated by the aforementioned methods without strictly identifying their subcellular origins. In line with the MISEV guidelines (154), the “exosomes” in these studies should be referred to as “small EVs”. Furthermore, different isolation procedures vary in their ability to enrich EV subpopulations. Size-exclusion chromatography (SEC) dilutes samples and has low selectivity for particles of similar sizes but offers gentler processing that better preserves vesicle integrity (155). When comparing results across different studies, extra caution is needed because the choice of these methodologies directly affects the composition of the resulting EV populations and the interpretation of their functions.

Given these considerations, this study uses terminology such as “exosomes” and “TDEs” consistent with the cited literature. However, readers should note that these terms refer to enriched populations of small EVs, whose activities result from the combined effects of mixed subpopulations. To advance this field toward higher standards of standardization, future research must incorporate more sophisticated isolation techniques, multiparametric characterization, and functional studies to better understand the unique roles of specific EV subtypes within the TME.

### 3.2 TME regulates exosomal secretion

In addition to acting as selective pressures for the adaptive survival of tumor cells, TMEal features such as hypoxia, inflammation, acidosis, and metabolic reprogramming serve as essential signaling sources that influence exosome biogenesis and release. This section will first examine how exosomes are regulated by two key microenvironmental signals: inflammation and hypoxia. We will then explore how three oncogenic signaling pathways (STAT3, Ras/Raf/MEK/ERK, and Wnt/β-catenin) affect exosome formation.

#### 3.2.1 Regulation by microenvironmental signals

Features of the TME, such as hypoxia, inflammation, and acidosis, exert a significant impact on exosome biosynthesis and release (106, 156). Hypoxia is one of the most potent stimuli in the TME, profoundly influencing exosome composition, function, and secretion (109, 157). Hypoxia-inducible factor 1α (HIF-1α) plays a key role in regulating the packaging of hypoxia-associated proteins and miRNAs into exosomes (158). In particular, hypoxia stimulates exosome release by upregulating Rab27a and downregulating Rab7. These exosomes enhance the therapeutic resistance, invasion, and migration of cancer cells.

Hypoxia induces the Warburg effect (aerobic glycolysis), and exosome secretion is directly associated with glycolytic activity; inhibiting glycolysis reduces exosome synthesis (159–161). Hypoxia also activates autophagy-related pathways in cancer-associated fibroblasts (CAFs), which promotes exosome release. These exosomes activate the NF-κB signaling pathway and increase matrix metalloproteinase 9 (MMP9) and interleukin-8 (IL-8) levels, leading to malignant progression in breast cancer cells (162). Hypoxic exosomes produced by glioblastoma cells can also induce angiogenesis (163). While the importance of HIF-1α is well-established, its specific molecular mechanisms remain unclear (164).

Inflammation is another crucial microenvironmental signal, as it stimulates tumor growth through a positive feedback loop involving exosomes. Tumor cells generate and release exosomes in response to inflammatory signals, for example, via the NF-κB pathway (165, 166). Exosomes can carry inflammatory signals: those harboring miR-27-3p can activate microglia, target peroxisome proliferator-activated receptor γ (PPARγ), and increase proinflammatory cytokines such as tumor necrosis factor-α (TNF-α), interleukin-1β (IL-1β), and interleukin-6 (IL-6), potentially exacerbating inflammation (167, 168). Exosomal miR-21 also promotes proinflammatory macrophage polarization by downregulating programmed cell death 4 (PDCD4) (169). Studies have shown that inhibiting relevant signaling pathways (including ROCK1) reduces exosome production, which in turn alleviates hepatic fibrosis and inflammation, indicating potential therapeutic strategies (170, 171).

#### 3.2.2 Signaling routes

Finally, intracellular signaling pathways must be integrated and implemented such that microenvironmental cues (such as hypoxia and inflammation) can regulate exosomes. Current research suggests that three well-known oncogenic pathways—STAT3, Ras/Raf/MEK/ERK, and Wnt/β-catenin—play a critical role in linking exosome formation to microenvironmental cues. The fact that these pathways are consistently activated in a range of tumor types, as well as their shown direct role in exosome cargo sorting, secretion regulation, and recipient cell functional alteration, justifies focusing on them. The mechanisms specific to each pathway are detailed in detail below.

##### 3.2.2.1 STAT3 signaling pathway

The STAT3 signaling pathway is a key regulator of the TME and is frequently constitutively activated in malignancies. Exosomes modulate the STAT3 pathway, influencing immune cell function and accelerating tumor growth through processes such as noncoding RNA transfer.

Exosomal miRNAs can target upstream inhibitors of STAT3. For example, exosomes derived from CRC contain miR-106a-5p, which inhibits suppressor of cytokine signaling 6 (SOCS6) to activate the JAK2/STAT3 pathway and induce M2 macrophage polarization (172). Certain miRNAs, such as exosomal miR-92b-3p/miR-1231-5p from bladder cancer, enhance STAT3 signaling by inhibiting phosphatase and tensin homolog (PTEN) and activating the PI3K/AKT pathway (173).

Tumor cells and other microenvironmental cells form positive feedback loops that continuously activate STAT3 via exosomes. For instance, in urothelial carcinoma, decreased speckle-type POZ protein (SPOP) expression in tumor cells increases STAT3 stability, which in turn induces the production of CCL2 to recruit macrophages. Following the secretion of IL-6 by these macrophages, STAT3 in tumor cells is further activated, forming a protumorigenic cycle (174). Cancer-associated fibroblast (CAF)-derived exosomes containing 6-sulfochondroitin sulfate induce the co-localization of STAT3 and GLI1 in macrophages, leading to the formation of an immunosuppressive TME. This simultaneously activates the Hedgehog and JAK/STAT3 pathways (175).

Interventions targeting the STAT3 pathway exhibit therapeutic potential. For example, PROTAC degraders, such as SD-36, selectively degrade STAT3 in DCs, enhancing anti-PD-1 efficacy and restoring antigen presentation function (176). The use of tumor-derived extracellular vesicles loaded with siSTAT3 and doxorubicin enables synergistic therapy through ICD and STAT3 gene silencing, which reverses TME immunosuppression (177).

##### 3.2.2.2 The Ras/Raf/MEK/ERK signaling pathway

The Ras/Raf/MEK/ERK signaling pathway, a major intracellular kinase cascade, precisely regulates exosome cargo sorting and secretion, exerting a profound impact on the TME.

Mutant KRAS in CRC selectively enhances the packaging of pro-metastatic miR-100 into exosomes while increasing the sorting of invasive proteins such as SRC and EGFR by activating the nSMase2-ceramide pathway. These exosomes enhance the invasiveness of recipient cells and contribute to the formation of pre-metastatic niches (178, 179).

Activated ERK1/2 facilitates PD-L1 sorting into exosomes by phosphorylating key components of the ESCRT complex, including the Hrs protein. By migrating to lymph nodes, PD-L1-bearing exosomes prevent CD8^+^ T cell exhaustion and confer resistance to anti-PD-1 therapy (180).

The MEK-ERK pathway regulates the tissue-specific expression of the RNA-binding protein Ago2. In CRC cells, phosphorylation of Ago2 reduces miRNA extrusion into exosomes, whereas in liver stem cells, it mediates Ago2 sorting via the Alix protein. This discrepancy suggests that the tissue microenvironment may influence the regulatory logic of this pathway regarding exosomal RNA cargo (181, 182).

The Ras/Raf/MEK/ERK signaling pathway also induces the transcriptional upregulation of the RNA-binding protein hnRNP H1. Meanwhile, hnRNP H1 upregulates secretory proteins and ESCRT components, promoting overall exosome secretion. Additionally, it facilitates A-Raf alternative splicing, which enhances Ras pathway activity. This positive feedback loop, which sustainably increases exosome secretion, accelerates tumor progression.

##### 3.2.2.3 The Wnt/β-catenin signaling pathway

The Wnt/β-catenin signaling pathway influences tumor growth, tissue regeneration, and exosome biogenesis and secretion through intercellular communication mediated by exosomes.

Wnt ligands, such as Wnt5b and Wnt3a, can be delivered to tumor cells (e.g., ovarian cancer cells) via exosomes derived from TAMs. This promotes cell proliferation and epithelial-mesenchymal transition (EMT) by activating the Wnt/β-catenin pathway, enhancing β-catenin nuclear translocation, and upregulating target genes such as c-Myc and Cyclin D1 (183–185). Activation of the Wnt/β-catenin pathway leads to increased exosome release: β-catenin-mediated nuclear translocation upregulates SNAP25 expression, facilitating multivesicular body (MVB)-plasma membrane fusion. By shifting the exocytosis-degradation balance, it inhibits the degradation of MVBs by lysosomes, thereby increasing exosome yield (186).

**Figure 3:**
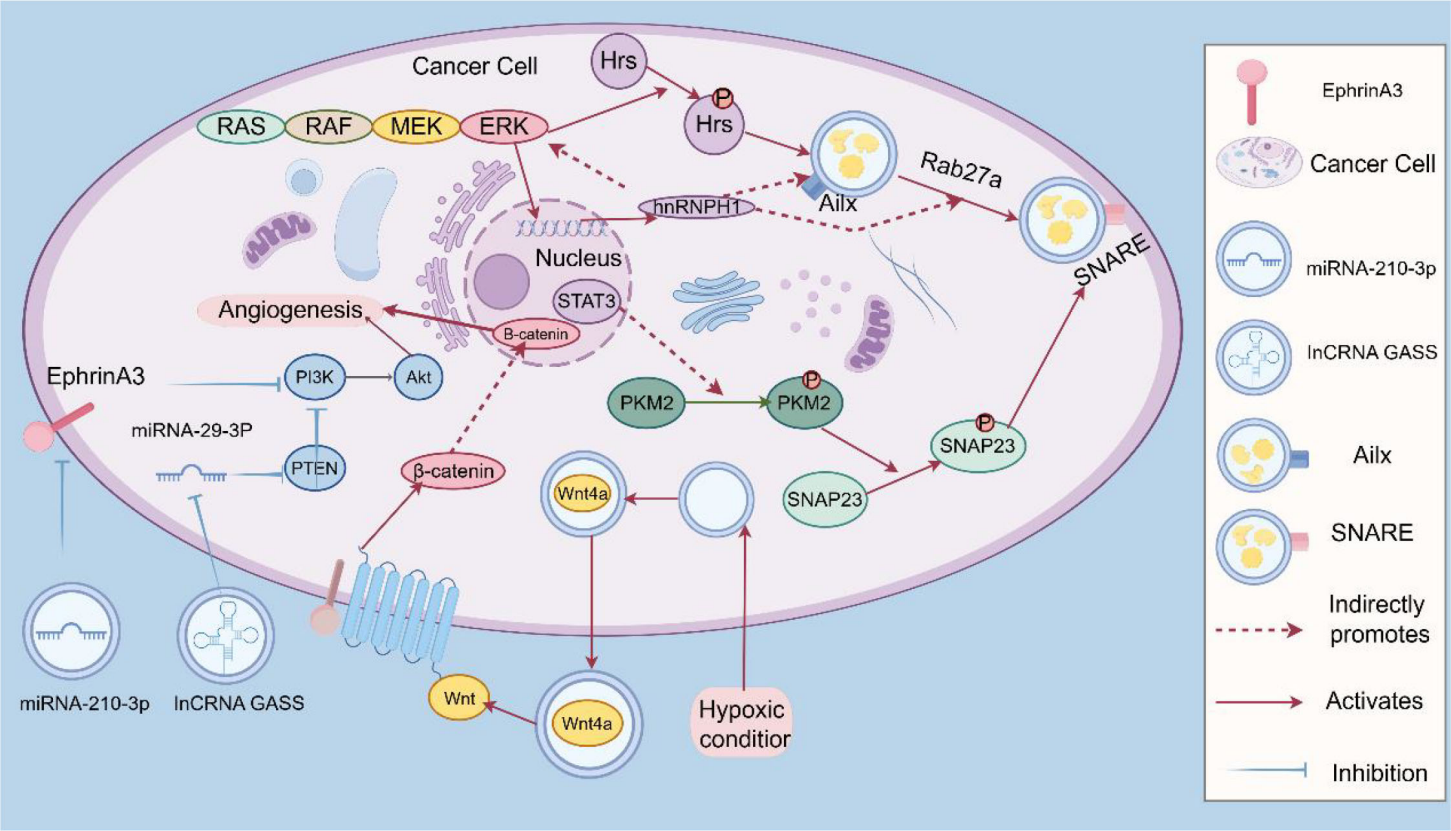
TME regulates exosome secretion.

Additionally, therapeutic strategies targeting the Wnt pathway have been investigated. Exosomes loaded with Wnt2 mRNA can target hepatic sinusoidal endothelial cells, activate Wnt/β-catenin signaling, alleviate drug-induced liver injury, and promote liver regeneration (187). (As illustrated in **Figure 3**).

Reading Pathway: Oncogenic signaling activates hnRNP H1, upregulates Alix/Rab27a, and promotes exosome release via SNAP23−mediated membrane fusion, ultimately inducing angiogenesis.

(This figure illustrates the mechanism by which oncogenic signaling regulates exosome secretion in the tumor microenvironment. Following activation of the classical RAS/RAF/MEK/ERK pathway in tumor cells, ERK−mediated signal transduction promotes the transcription of hnRNP H1, which further upregulates the expression of Alix and Rab27a, thereby enhancing exosome biogenesis. Meanwhile, a positive feedback loop exists between Ras signaling and hnRNP H1, and ERK−mediated Hrs phosphorylation further promotes exosome secretion. These exosomes are released via SNAP23−mediated membrane fusion and, upon uptake by recipient cells, activate key signaling pathways including PI3K/Akt and Wnt/β−catenin, driving transcription factors such as STAT3 into the nucleus to regulate gene expression, thereby effectively inducing angiogenesis to support tumor growth. This figure also highlights the critical roles of the hypoxic tumor microenvironment and membrane fusion proteins such as SNAP23 in the regulatory process of exosome secretion, collectively demonstrating the core function of exosomes as intercellular communication mediators in tumor development).

### 3.3 Exosome-mediated TME remodeling

TDEs systematically reprogram the functions of various cells within the TME by delivering bioactive molecules such as proteins and nucleic acids, thereby creating a microenvironment conducive to tumor growth, invasion, and metastasis (188, 189). Extensive research has confirmed that TDEs comprehensively drive tumor progression by promoting EMT in tumor cells, inducing angiogenesis, establishing pre-metastatic niches, and mediating immune evasion (190, 191). This section will focus on the regulatory effects of exosomes on two core cell types—T lymphocytes and macrophages—followed by an analysis of their protumorigenic functions in tumor angiogenesis. Finally, we will summarize the antitumor potential of engineered exosomes in reversing immune suppression.

#### 3.3.1 Tumor-promoting effect

The tumor immune process is a multifaceted and dynamic phenomenon involving various immune cells and bioactive molecules within the body. Tumor cells employ multiple strategies to evade the host immune response, including suppressing tumor antigens, inhibiting regulatory immune cells, and secreting immunosuppressive molecules (188). Within tumor tissues, communication between tumor cells and immune cells occurs via exosomes, which create an environment conducive to tumor growth (189). Studies confirm that tumor-derived exosomes promote tumor progression by guiding metastasis through facilitating EMT in tumor cells, inducing angiogenesis, establishing pre-metastatic microenvironments, enabling immune evasion, forming metastatic niches, and influencing organ tropism.

##### 3.3.1.1 Inhibition of T cell activity

T lymphocytes serve as the core effector cells in antitumor immunity. However, TDEs can directly suppress their function through multiple mechanisms, thereby inducing an immunosuppressive TME. TDEs deliver specific circular RNAs (circRNAs) into T cells, where they act as molecular sponges to regulate gene expression, directly leading to T cell dysfunction or exhaustion. In HCC, exosomal circCCAR1 enters CD8^+^ T cells and stabilizes PD-1 expression, resulting in functional impairment and drug resistance (192). In non-small cell lung cancer (NSCLC), exosomal circUSP7 inhibits IFN-γ and TNF-α secretion by CD8^+^ T cells via the miR-934/SHP2 axis (193). In lung adenocarcinoma, exosomal circRNA-002178 simultaneously upregulates PD-L1 on tumor cells and PD-1 on T cells, synergistically inducing CD8^+^ T cell exhaustion through dual pathways (194).

Immunomodulatory proteins carried on the surface of TDEs can directly interact with T cells to transmit inhibitory signals. Exosomes expressing PD-L1 bind to PD-1 on T cells, suppressing their activation and cytokine secretion while contributing to immunotherapy resistance (195–197). Exosomes displaying FasL directly trigger apoptosis in CD8^+^ T cells (198).

TDEs also act on other immune cell populations to indirectly suppress T cell function. They induce the proliferation and activation of MDSCs via HSP72, which in turn inhibits T cell activity (199, 200). Additionally, surface-expressed CD39/CD73 ectoenzymes hydrolyze ATP into adenosine, establishing a chemical barrier within the TME that suppresses both T and B lymphocytes (200, 201).

##### 3.3.1.2 Induction of M2 macrophage polarization

Exosomal circRNAs have been shown to regulate macrophage M1/M2 polarization, thereby promoting tumor progression and immune evasion (202). For instance, in renal cell carcinoma, exosomal circSAFB2 alleviates the inhibition of the JAK1/STAT3 pathway by sponging miR-620, driving M2 polarization and facilitating tumor metastasis and immune escape (203). In glioma, hnRNPA2B1 packages circNEIL3 into exosomes, enhancing the immunosuppressive function of infiltrating TAMs (204). Under endoplasmic reticulum stress in breast cancer, exosomal circ_0001142 targets the miR-361-3p/PIK3CB pathway to induce M2 polarization and suppress macrophage autophagy (205). Similarly, under hypoxic conditions in esophageal cancer, hypoxia-associated exosomal circ0048117 directly drives macrophage M2 conversion (206). Furthermore, exosomes derived from lung cancer cells deliver TRIM59 and promote the proteasomal degradation of ABHD5, converting macrophages into a tumor-promoting phenotype. This process activates the NLRP3 inflammasome signaling pathway and promotes tumor progression via IL-1 secretion (207, 208).

Hypoxia within the TME serves as a critical driver of exosome-dependent macrophage reprogramming. In glioma models, hypoxia triggers tumor cells to secrete exosomes enriched with IL-6 and miRNA-155-3p. Following uptake by macrophages, IL-6 upregulates miRNA-155-3p expression via activation of the STAT3 signaling pathway, subsequently inducing macrophage autophagy. This autophagic process further enhances STAT3 phosphorylation, thereby potentiating tumorigenesis. Exosome-induced autophagy ultimately leads to M2 polarization of macrophages and facilitates accelerated glioma progression (209).

##### 3.3.1.3 Promotion of angiogenesis

Tumor-derived exosomes play a pivotal role in promoting tumor angiogenesis by delivering a variety of bioactive molecules, thereby supporting tumor growth and metastasis. Multiple tumor-derived exosomal circRNAs have been shown to induce angiogenesis directly or indirectly via regulating associated signaling pathways. In gastric cancer, exosomal circ29 relieves the inhibition on vascular endothelial growth factor (VEGF) by sponging miR-29a, upregulating VEGF expression and activating endothelial cells (210). In hepatocellular carcinoma, exosomal circRNA-100338 activates the mTOR/HIF-1α pathway via the miR-141-3p/RHEB axis, promoting VEGF expression and angiogenesis (211). In glioblastoma, microglia-derived exosomal circKIF18A enhances the angiogenic activity of human brain microvascular endothelial cells (212).

Exosome-carried miRNAs participate in the regulation of angiogenesis by influencing endothelial cell function. Lung cancer-derived exosomal miR-23a leads to HIF-1α accumulation by inhibiting PHD1/PHD2, thereby enhancing angiogenesis; simultaneously, it increases vascular permeability by suppressing ZO-1, facilitating tumor cell migration (213). Exosomal miR-21 upregulates VEGF via STAT3 pathway activation, promoting angiogenesis and malignant transformation (214); miR-126 has also demonstrated pro-angiogenic effects in non-small cell lung cancer (215).

Hypoxia enhances the pro-angiogenic capacity of exosomes derived from lung cancer stem cells, whereas aspirin attenuates this effect by altering exosomal components, including upregulation of HIF-1α/COX-2 and downregulation of miR-135b and miR-210 (216). Low levels of soluble fms-like tyrosine kinase 1 (sFlt-1) in small cell lung cancer exosomes promote endothelial cell migration, suggesting that sFlt-1 may possess potential to inhibit metastasis (217).

Collectively, exosomes directly or indirectly regulate key angiogenic factors—such as VEGF and HIF-1α—through the delivery of circRNAs, miRNAs, and other molecular mediators, thereby promoting tumor neovascularization. Targeting exosome-mediated angiogenic pathways may offer novel strategies for antitumor therapy.

#### 3.3.2 Tumor-suppressive function

Engineered exosomes offer a novel strategy for reversing the immunosuppressive TME and enhancing antitumor efficacy. The core strategy involves the precise delivery of specific functional molecules into the TME through rational design, thereby reprogramming immune cells or directly eliminating tumor cells.

M2-type TAMs represent the predominant immunosuppressive cell population within the TME. Studies have utilized engineered exosomes loaded with STAT6 antisense oligonucleotides to achieve efficient targeted delivery to M2-type macrophages. This strategy successfully reprograms TAMs from the immunosuppressive M2 phenotype toward the pro-inflammatory, antitumor M1 phenotype, significantly activating CD8^+^ T cell immune responses. This approach achieved over 90% tumor growth inhibition in experimental models, providing a novel avenue for overcoming immune tolerance (218, 219).

Engineered exosomes can act as sensitizers to enhance the efficacy of conventional therapies such as radiotherapy. For instance, manganese carbonyl-loaded exosomes catalyze the generation of abundant ROS within the TME, and in combination with low-dose radiotherapy, exert potent inhibition of tumor proliferation, markedly improving therapeutic outcomes (220).

By leveraging TME characteristics—such as weak acidity and high expression of specific enzymes—”smart” responsive exosomes can be designed. For example, exosomes engineered to respond to the inflammatory TME efficiently cross the blood-brain barrier and precisely deliver chemotherapeutic agents such as doxorubicin to glioma regions, effectively suppressing tumor progression (221).

In summary, engineered exosomes open promising avenues for reshaping the TME and developing next-generation anticancer therapies through precision targeting, immune cell reprogramming, and synergistic enhancement of therapeutic efficacy.

### 3.4 Clinical challenges of the TME-ICD interplay

As discussed above, the intricate interplay between the TME and exosomes plays a central role in tumor initiation and progression. However, translating these fundamental insights into clinical practice still faces multiple challenges, ranging from technical standardization to mechanistic understanding. This section systematically analyzes these challenges from three perspectives: the current status and pathways of clinical translation, the limitations of theoretical frameworks and insights from negative findings, and future directions and prospects. This analytical framework aims to provide a clear perspective for reflection and a roadmap for future investigations.

#### 3.4.1 Clinical translation of the TME-exosome axis

The interactive framework between the TME and exosomes is rapidly advancing from basic research toward clinical application, with core pathways spanning three major dimensions: diagnosis, treatment, and monitoring.

In the field of liquid biopsy, tumor-derived exosomes—due to their carriage of parent cell-specific proteins, nucleic acids, and lipids, as well as their stable presence in body fluids—are hailed as “molecular avatars” that reflect tumor dynamics (151). Compared with traditional tissue biopsy, exosome-based assays offer advantages including minimal invasiveness, feasibility of serial sampling, and the ability to overcome tumor heterogeneity. Clinical studies have shown that exosomal integrin expression profiles can prospectively identify patients at high risk of organ-specific metastasis—for instance, α6β4 integrin predicts liver metastasis, whereas αvβ5 is associated with pulmonary dissemination (154). Furthermore, dynamic changes in exosomal PD-L1 levels can reflect the response status to immune checkpoint inhibitors earlier than radiographic assessments, providing a rationale for real-time therapeutic adjustments (222).

At the therapeutic level, exosomes present dual application prospects. On one hand, targeted inhibition of protumor exosome biogenesis—through interventions such as Rab27a or nSMase2 blockade—can disrupt malignant signal transmission within the TME. Preclinical models have demonstrated the potential of this approach to suppress tumor progression and sensitize tumors to chemotherapy (223). On the other hand, engineered exosomes are gradually entering clinical evaluation as novel drug delivery platforms. For example, CAR-T cell-derived exosomes retain cytotoxic activity while circumventing the risk of cytokine release syndrome (151). To date, at least four targeted therapies based on extracellular vesicles have entered Phase I clinical trials.

Regarding therapeutic monitoring, exosomes provide technical support for full-course management through their capacity to dynamically reflect TME alterations. By tracking exosomal markers such as PD-L1 and miR-21, real-time assessment of drug sensitivity and the timing of resistance acquisition can be achieved (154). The development of 3D culture models and organoid technologies has further enhanced the clinical predictive value of exosome research, facilitating the discovery of biomarkers with greater translational potential (224).

#### 3.4.2 Theoretical limitations and insights from negative findings

Despite the increasingly refined framework of TME-exosome interactions, current research still faces multiple limitations, with certain negative findings offering critical insights for advancing the field.

Limitations of experimental models and heterogeneity challenges. The majority of studies rely on 2D culture systems, which fail to recapitulate the cell-matrix interactions, oxygen gradients, and metabolic heterogeneity characteristic of the *in vivo* TME. Notably, acellular factors such as pH and pO_2_ exert significant influence on exosome biogenesis and composition (225). Furthermore, exosomes exhibit substantial heterogeneity in size, cellular origin, and cargo composition. According to the MISEV2023 guidelines, size-based classification alone is insufficient to define distinct exosome subpopulations (151).

Mixed outcomes from exosome inhibition strategies. Early studies anticipated that inhibiting exosome biogenesis could disrupt malignant signaling; however, preclinical models have yielded inconsistent results with inhibitors alone (223). Since exosomes are secreted by multiple cell types and participate in maintaining tissue homeostasis, non-selective inhibition may disrupt normal intercellular communication. This necessitates the development of selective strategies targeting tumor-specific exosome subpopulations or blocking the packaging of specific pathogenic cargoes. Moreover, the application of exosome inhibitors may be accompanied by off-target effects (226).

Context-dependent functions of exosomes from different cellular origins. Exosomes derived from distinct cell sources within the TME may exert opposing functions. For instance, the functional impact of MSC-derived exosomes depends critically on the inflammatory status of the parent MSCs (227). Donor variability and tissue source heterogeneity contribute to batch-to-batch inconsistencies, directly compromising therapeutic reproducibility (228).

Barriers to clinical translation of engineered exosomes. Despite promising preclinical results, engineered exosomes face multiple obstacles in clinical application: lack of standardized protocols for scalable production, inconsistencies in purification methods leading to batch variability, and unresolved questions regarding long-term safety. The U.S. Food and Drug Administration (FDA) has already issued public safety warnings regarding exosome-based products (229).

In summary, the value of negative findings lies in their reminder that exosome research must transition from descriptive observations to mechanistic understanding, from single-center positive reports to multicenter validation, and from broad-brush interventions toward precision targeting.

#### 3.4.3 Clinical implications and future perspectives of the TME-exosome interplay

The deepening understanding of the TME-exosome interaction framework is reshaping oncology knowledge and paving new pathways for precision medicine.

Viewing the TME and exosomes as a dynamic interactive system implies that tumor treatment must shift from a single-point mindset of “killing tumor cells” to a systemic approach of “reshaping the tumor ecosystem” (230). This framework suggests that effective intervention must simultaneously address three aspects: suppressing tumor cells’ release of pro-cancer signals, blocking exosome pre-conditioning of distant microenvironments, and enhancing the anti-tumor activity of immune cell-derived exosomes (231).

Based on this framework, future therapies will trend toward combination approaches, such as targeting Rab-dependent vesicle transport proteins like Rab27a and nSMase2 (232); neutralizing functional molecules on exosome surfaces (e.g., PD-L1) via antibodies or disrupting receptor endocytosis pathways (233); utilizing engineered exosomes to deliver immune agonists or CRISPR/Cas9 systems to reshape the immune microenvironment (234); and combining exosome interventions with immune checkpoint inhibitors, chemotherapy, or radiotherapy to overcome treatment resistance.

From mechanism-diagnosis-treatment closed-loop management to precision stratification. The ultimate vision of exosome research is to achieve closed-loop management throughout the entire tumor disease course (235), such as detecting high-risk exosome biomarkers in the early stages via liquid biopsy (236); adjusting treatment regimens based on dynamic changes during therapy (237); and monitoring minimal residual disease and providing early warnings for post-treatment resistance (235). Achieving this goal requires overcoming two major challenges: establishing standardized exosome isolation and detection protocols (238); and developing multi-omics technologies with single-vesicle resolution to decipher the information complexity arising from tumor heterogeneity and exosome subtype diversity (239).

**Figure 4:**
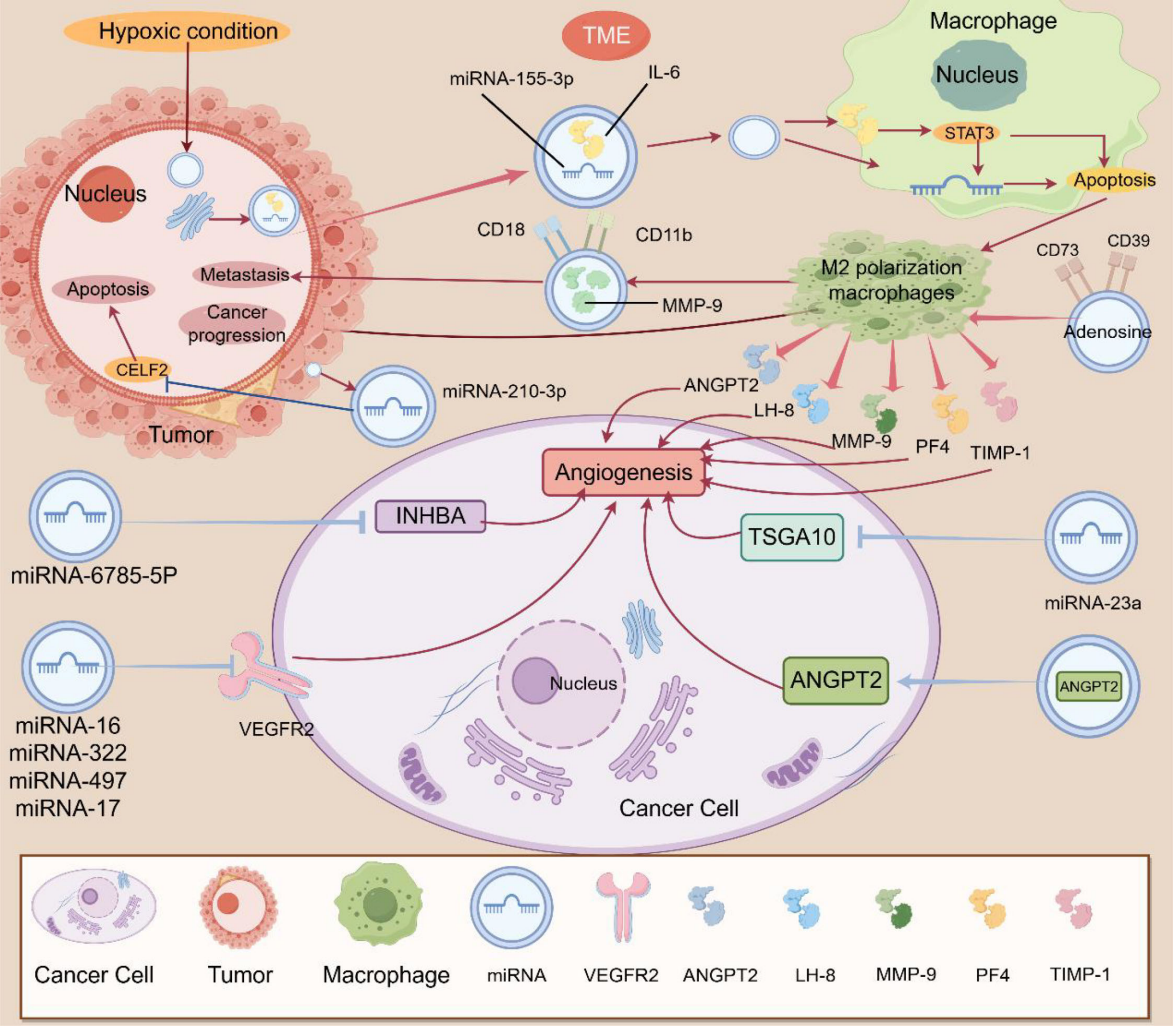
Exosome-mediated TME remodeling.

Looking ahead, the field must focus on breakthroughs in the following areas: mapping the exosome landscape across cell types within the TME to elucidate the functional roles and interaction networks of distinct subpopulations (231); deciphering the molecular mechanisms and regulatory networks governing exosomal cargo sorting to identify actionable therapeutic targets (239); advancing innovative clinical trial designs that utilize exosomal biomarkers as enrichment strategies and surrogate endpoints to accelerate the clinical translation of effective interventions (237). (As illustrated in **Figure 4**; **Table 4**).

**Table 4:** Key intervention strategies and drugs targeting the TME–exosome crosstalk.

Strategies/agents	Mechanism of action	Research model	Evidence of exosome regulation	Immunological/therapeutic outcomes	References
PROTAC degrader (SD-36/SD-2301)	Selectively degrades STAT3 in DCs, restoring their antigen presentation function	Mouse tumor model	Targets the STAT3 pathway, indirectly modulating exosome-mediated immune regulation	Enhances anti-PD-1 efficacy; activates CD8^+^ T cell responses	(176)
Tumor-derived extracellular vesicles (TEV)	Encapsulate siSTAT3 and doxorubicin (DOX), silencing the STAT3 gene and inducing ICD	Mouse tumor model	Utilizes exosomes as delivery vehicles to achieve synergistic therapy via STAT3 gene silencing and ICD	Reverses TME immunosuppression; inhibits tumor growth	(177)
nSMase inhibitor (GW4869)	Blocks mutant KRAS-dependent secretion of miR-100-containing exosomes	Mouse model of CRC	Inhibits nSMase2, a key enzyme in exosome biogenesis, thereby reducing packaging of pro-metastatic miRNAs into exosomes	Inhibits pre-metastatic niche formation; reduces tumor invasiveness	(178)
Zoledronic acid (ZA)	Inhibits M2-like TAM polarization and the Wnt/β-catenin pathway	Mouse tumor model	Modulates Wnt signaling activation in tumor cells mediated by TAM-derived exosomes	Suppresses tumor cell proliferation and metastasis	(185)
Aspirin	Reduce exosome secretion and alter exosome composition (upregulate HIF-1α/COX-2, downregulate miR-135b and miR-210)	Lung cancer stem cell model	Remodels exosomal cargo composition; attenuates hypoxia-induced pro-angiogenic capacity	Inhibits angiogenesis; reduces metastatic potential	(216)
Manganese carbonyl-loaded exosomes	Targets delivery to TME; catalyzes burst of ROS generation	Mouse tumor model	Engineered exosomes serve as drug delivery platform for manganese carbonyl loading	Combined with radiotherapy inhibits tumor proliferation	(220)
Doxorubicin-loaded exosomes	Crosses the blood-brain barrier (BBB), efficiently delivering doxorubicin to the glioma TME	Mouse model of glioma	Engineered exosomes achieve precision drug delivery across physiological barriers	Effectively suppresses tumor progression	(221)
STAT6 antisense oligonucleotide (ASO)-loaded exosomes	Enhances delivery efficiency compared to free ASO; inhibits STAT6 pathway gene expression	Mouse tumor model	Engineered exosomes achieve targeted ASO delivery to M2-type TAMs	Induces TAM repolarization from M2 to M1 phenotype; activates CD8^+^ T cell immune responses (tumor inhibition rate >90%)	(218, 219)
Anti-PD-L1 antibody	Neutralizes PD-L1 on exosome surfaces; blocks its inhibitory signaling to T cells	Preclinical model	Targets functional molecules on exosome surfaces; interferes with their interaction with PD-1 on T cells	Restores T cell activation; reverses immunotherapy resistance	(195–197)

Reading Pathway: Hypoxia promotes the secretion of exosomes carrying IL-6 and miR-155, which drive macrophage M2 polarization, angiogenesis, and metastasis.

(This figure illustrates how hypoxia, as a core feature of the TME, drives tumor cells to remodel the TME through exosome-mediated communication networks. Hypoxic conditions induce cancer cells to se exosomes, which subsequently trigger autophagy and promote M2 polarization of macrophages, thereby facilitating cancer progression. These exosomes contain high levels of IL-6 and miR-155-3p. STAT3 activation occurs via IL-6, which in turn enhances miR-155-3p expression to induce autophagy. Exosomes influence macrophage polarization, and M2-polarized macrophages secrete multiple factors, including ANGPT2 and MMP-9, that participate in TME regulation. Hypoxia also promotes the secretion of exosomes enriched with miR-210-3p within the TME, which inhibit cell death and promote G1-to-S phase transition by downregulating CELF2 expression. Additionally, various miRNAs, including miR-6785-5p and miR-16, modulate tumor cell function by targeting molecules such as INHBA and VEGFR2. Exosome-carried molecules including ANGPT2 and TSGA10, together with miRNA-regulated molecules such as INHBA in tumor cells, collectively drive angiogenesis, supporting tumor growth and dissemination, thereby influencing tumor progression and metastasis).

## 4 The interaction framework between extracellular vesicles and ICD

EVs facilitate intercellular communication, and their cargo can regulate cellular functions, thereby influencing the activity and functional phenotypes of tumor cells and immune cells. ICD, also referred to as immune-stimulatory cell death, is a highly immunogenic form of cell death. Inflammatory molecules released during ICD, such as DAMPs, typically activate immune cells to exert antitumor effects. The crosstalk between these two processes (EVs and ICD) plays a critical role in regulating tumor immunity. Therefore, this chapter first examines the effects of ICD on extracellular vesicles, then analyzes the extracellular vesicle-mediated regulation of ICD and the extracellular vesicle-ICD-dependent intercellular communication network, and concludes with the translational framework of extracellular vesicle-ICD interactions. By elucidating the intricate relationship between these two processes, we aim to provide valuable insights for the development of therapeutic strategies involving exosomes and ICD. **(As illustrated in****Table 5**).

**Table 5:** Interaction framework between exosomes and ICD.

Interaction framework between exosomes and ICD	Primary regulatory aspects	Key factors/mechanisms
Impact of Immunogenic Cell Death on Extracellular Vesicle Biology	Extracellular vesicles as amplifiers of ICD signaling	Extracellular vesicles mediate the delivery of DAMPs and nucleic acids, regulating antitumor immunity, among other functions.
	Mechanisms by Which Extracellular Vesicles Suppress Immune Responses	Immune checkpoint molecules and their functions, as well as epigenetic regulation, etc.
	Heterogeneity in Extracellular Vesicle Origins Influences ICD	Differential Effects of Tumor-Derived and Immune-Cell-Derived Extracellular Vesicles on Modulating Immune Responses
Extracellular Vesicle-Mediated ICD Effects	Engineered EVs modulating ICD	Modified extracellular vesicles induce programmed cell death for antitumor immunity
Extracellular Vesicle-ICD-Mediated Intercellular Communication Network	Tumor cell-immune cell crosstalk	Tumor-derived EVs modulating DC function
	Interactions between immune cells	Macrophage polarization; NK cell activation
Translational Framework for Extracellular Vesicles and ICD Interaction	Challenges in Combining Extracellular Vesicles with Induced Cell Death for Antitumor Therapy	The tumor-promoting effects of extracellular vesicle contents and the challenges in applying engineered extracellular vesicles
	Clinical translation and future directions	Clinical Efficacy and Future Development Directions of Engineered Extracellular Vesicles in Cancer Therapy

### 4.1 Impact of immunogenic cell death on extracellular vesicle biology

#### 4.1.1 Extracellular vesicles as amplifiers of ICD signaling

##### 4.1.1.1 Extracellular vesicles mediate the delivery of DAMPs

DAMPs are a class of endogenous cellular molecules that activate the immune system, including DNA, RNA, HSPs, CRT, ATP, and HMGB1 (240–242). When cells undergo stress or injury, DAMPs can be released into the extracellular space (240). The release of DAMPs serves as one of the key features of ICD, effectively activating immune responses (243). EVs can encapsulate DAMPs internally or carry them on their membranes for secretion via exocytosis, and this release is enhanced under stress conditions (244).

HSP70 is a molecular chaperone involved in protein synthesis, folding, repair, and degradation, as well as assisting in protein transport to organelles. When released extracellularly, HSP70 exhibits strong immunogenicity. However, in contexts such as cancer, HSP70 promotes tumor progression and drug resistance (245). Broquet et al. found that following treatment with brefeldin A or monensin, HSP70 translocates to the plasma membrane. Membrane-associated heat shock protein 70 (mHSP70) localizes to lipid rafts and can form complexes (246). The secretion of soluble HSP70 (sHSP70) depends on the endosomal-lysosomal pathway and is associated with lysosomal-associated membrane protein 1 (LAMP1) (247). HSP70 can also enter lysosomes via ATP-binding cassette (ABC) transporter family proteins expressed on endosomes (247).

It is currently believed that HSPs can be released via EVs such as microvesicles (248). HSPs can activate various immune cells to induce immune responses. Among these cells, T cells, NK cells, macrophages, and DCs can exert corresponding immune functions following activation by HSPs (122). Within the TME, HSP70 secreted by cancer cells interacts with toll-like receptor 2 (TLR2), inducing M2 macrophage polarization. Therefore, targeted disruption of the HSP70-TLR2-MerTK interaction may alleviate immune suppression in the TME (249). Furthermore, EVs from heat-stressed tumor cells (HS-TEXs) are enriched with HSP70, which not only stimulates antitumor immune responses but also promotes IL-6 secretion by DCs to antagonize TGF-β1-induced Treg differentiation (250).

HMGB1, by virtue of its polybasic regions and protein-transduction-like domain structure, is released extracellularly via lysosomal exocytosis and passive diffusion (251, 252). HMGB1-containing EVs from MP cells can induce pro-inflammatory phenotype conversion in macrophages and epithelial cells, contributing to disease pathogenesis (253). Concurrently, HMGB1-carrying EVs may also act on monocytes or DCs to sustain systemic inflammatory responses. Beyond sustaining chronic inflammation and promoting wound healing, HMGB1 facilitates tumor progression via angiogenesis, reparative epithelial proliferation, and immune suppression mediated by MDSCs and Tregs (254). HMGB1 activates specific PRRs, including TLRs, which promote tumor metastasis, immune evasion, and neovascularization (254). HMGB1-containing EVs from HCC cells induce regulatory B (Breg) cell differentiation, proliferation, and activation, thereby contributing to immunosuppression (255). Conversely, miR-142-3p expression is upregulated in EVs from M1 macrophages, where it targets HMGB1 to inhibit glioblastoma (GBM) cell growth (256). Furthermore, 5-aminolevulinic acid photodynamic therapy (ALA-PDT) modulates miR-34a-mediated HMGB1 secretion via EVs, inhibiting the proliferative capacity of HPV-positive cervical cancer cells while promoting their apoptosis (257).

The aforementioned regulatory mechanisms between DAMPs and EVs reveal that this interaction can be harnessed to improve clinical tumor therapy outcomes. Specifically, EVs can be artificially induced or engineered to carry immunostimulatory DAMPs such as HSP70 to awaken the immune system. Concurrently, reducing or blocking the secretion of EVs carrying immunosuppressive DAMPs (e.g., HMGB1) can alleviate immunosuppressive TMEs.

##### 4.1.1.2 Extracellular vesicles mediate the delivery of nucleic acids

EVs are nanoscale vesicles that participate in intercellular communication and regulate various biological processes. TEXs can carry genetic material including DNA and miRNA, acting as signaling molecules for intercellular transmission and transport (258). miRNAs can undergo functional intercellular transfer via EVs and exert their regulatory effects (259). As miRNA carriers, EVs facilitate intercellular communication and information exchange through paracrine/endocrine pathways, playing crucial roles in tumor growth, angiogenesis, and immune suppression. miRNA levels within EVs can fluctuate in response to stress conditions. Under certain circumstances, specific miRNAs bind to TLRs, acting as danger signals that modulate the activity of immune cells such as NK cells within the TME (99). TEXs encapsulating miRNAs deliver biological information and facilitate cellular communication with neighboring cells, thereby promoting the establishment, maintenance, and enhancement of the cancer TME and metastatic niches (260).

Cancer-derived miRNA-containing EVs participate in the recruitment and reprogramming of cellular components within the TME (261), thereby promoting the malignant transformation and metastasis of cancer cells. EV-derived miR-21 and miR-155 mediate the transmission of chemoresistance between neuroblastoma cells and human monocytes via the EV miR-21/TLR8–NF-κB/EV miR-155/TERF1 signaling axis (262). NSCLC secretes EVs enriched in miR-21 and miR-29a, which bind to TLRs and trigger prometastatic inflammatory responses, ultimately promoting tumor growth and metastasis (263). Transformed human bronchial epithelial (HBE) cells transfer miR-21 to normal HBE cells via EVs, activating the STAT3 signaling pathway and upregulating VEGF expression, thereby contributing to angiogenesis and malignant cellular transformation (214). In addition, stromal cells release RNA-containing EVs that elicit antiviral signaling in recipient breast cancer cells, ultimately accelerating tumor growth (264). miRNAs can undergo functional intercellular transfer via EVs, and vesicular miRNA profiles can be remodeled in response to stress conditions. Specific miRNAs that bind to TLRs can activate innate immune cells (99). These findings have also inspired novel therapeutic strategies. HA nanoparticles loaded with miR-125b promote M1 macrophage polarization and enhance antitumor efficacy (265). Systemic delivery of plasmid DNA using CD44/EGFR dual-targeting nanoparticles (NPs) increased wild-type p53 (wt-p53) and miR-125b expression in SK-LU-1 human lung adenocarcinoma cells. This miR-125b/wt-p53 plasmid system induced M1 polarization and suppressed tumor growth (266).

As outlined above, EV nucleic acids exert dual functions in tumor progression. While EV nucleic acids derived from tumor cells frequently promote tumor progression, stress stimuli can remodel their cargo composition to modulate immune cell function toward antitumor activity. Therefore, applying ICD inducers to trigger stress in cancer cells may redirect the function of their EV nucleic acids toward antitumor immunity, representing a promising strategy for clinical immunotherapy.

#### 4.1.2 Mechanisms by which extracellular vesicles suppress immune responses

##### 4.1.2.1 Extracellular vesicles shape the suppressive immune microenvironment through immune checkpoint molecules

Tumor-derived extracellular vesicles can carry immunosuppressive signals to inactivate effector cells and facilitate tumor immune escape while also participating in processes such as angiogenesis and microenvironment remodeling (267). PD-L1 within extracellular vesicles functions as an immunosuppressive molecule, effectively inhibiting the activity of immune cells, including CD8^+^ T cells (268), thereby promoting tumor progression and metastasis. Research indicates that inhibiting PD-L1 signaling in extracellular vesicles attenuates their immunosuppressive effects and enhances antitumor immune responses (269). Analysis of clinical samples reveals detectable PD-L1 on the surface of extracellular vesicles derived from HNSCC patient plasma, with expression levels correlated to disease progression, UICC staging, and lymph node status (269). Furthermore, immune checkpoint molecules such as cytotoxic T-lymphocyte-associated antigen 4 (CTLA-4) and PD-L2 are elevated in plasma extracellular vesicles from HNSCC patients, accompanied by increased macrophage type 2 polarization and CXCL4 secretion (270).

CTLA-4 is a readily inactivated membrane protein expressed on the surface of activated T cells. It can be internalized into the cell, dissociate from antibodies within the endosome, and subsequently recycle back to the cell membrane. CTLA-4 suppresses the response of resting T cells, a function dependent on its expression level and the number of APCs. CTLA-4 also captures the CD80 ligand on adjacent APC surfaces, transporting it into lysosomes for degradation, thereby attenuating co-stimulatory signals (271, 272). CTLA-4 binds to CD28 on T cells and interacts with CD80 and CD86 on APCs during T cell activation, transmitting inhibitory signals to T cells (273). Upregulation of CTLA-4 expression in extracellular vesicles modulates the PTEN/CD44 pathway to promote hepatocellular carcinoma invasion and metastasis (273). Conversely, the endosomal deubiquitinating enzyme USP8 interacts with CTLA-4; USP8 deficiency enhances CTLA-4 ubiquitination in extracellular vesicles derived from both CD4^+^ T cells and cancer cells, thereby releasing T cell activation inhibition (274).

In summary, immune checkpoint molecules such as PD-L1 and CTLA-4, when delivered to T cells via extracellular vesicles, accelerate T cell exhaustion and suppress their function. By inhibiting tumor cells to reduce secretion of these extracellular vesicles, or by targeting PD-L1 and CTLA-4 within the vesicles to neutralize their function, T cell anti-tumor activity can be restored. This approach may improve clinical resistance to tumor immunotherapy.

##### 4.1.2.2 Epigenetic regulation: histone modifications, DNA methylation

Histones constitute a significant component of the proteome in various EVs, including microvesicles, apoptotic vesicles, and exosomes (275). Core histones and linker histones localize within EVs and are secreted extracellularly via the MVB/EV pathway. Under cellular stress conditions, histone secretion into EVs is upregulated, accompanied by increased vesicle numbers and a shift toward smaller-sized EVs (275). Tamoxifen treatment upregulates miR-329 expression in EVs from MCF-7 cancer cells, thereby inhibiting histone demethylase KDM1A, reducing angiogenesis, and exerting antitumor effects (276). Circ0088494 derived from M2-type EVs recruits histone methyltransferase KMT2D, promoting H3K4 monomethylation at the STEAP3 gene locus to inhibit ferroptosis in skin squamous cell carcinoma (277). In PIK3CA-mutant tumors, arachidonic acid transported via EVs to intestinal epithelial cells binds to menin, enhancing its interaction with the MLL1/2 methyltransferase and promoting malignant transformation of intestinal epithelial cells (278).

EVs can also carry methylation regulators such as DNA methyltransferases (DNMTs) and demethylases (TETs), delivering methylated DNA fragments to shape immunosuppressive microenvironments and promote cancer immune escape. EVs can transfer DNMT mRNA to recipient monocytes, which exhibit significant methylation changes and gene suppression, inducing an immunosuppressive phenotype in human monocytes (279). Bone marrow-derived MSCs drive the dedifferentiation of breast cancer cells into cancer stem cells by transferring histone methyltransferases KMT2B/D and DNMTs via EVs (280). DNA methyltransferases DNMT3A and DNMT3B suppress the expression of the candidate tumor suppressor miR-493-5p, thereby diminishing its EV secretion and anti-angiogenic effects, and promoting tumor proliferation and invasion (281). Methylation patterns and structural features of certain tumor-derived circulating cell-free DNA (cfDNA) fragments reflect chromatin activity in cancer cells. Decoding cfDNA epigenetic characteristics holds promise for early cancer detection (282).

The regulatory role of EVs in epigenetics is primarily manifested through “molecular packaging” and “long-range regulation,” enabling cross-cell epigenetic reprogramming. This process reshapes recipient cells such as tumor cells, enhancing their malignant characteristics. However, pre-treating tumor-derived EVs with epigenetic drugs to alter their contents could transform them into “endogenous tumor vaccines.” This approach may suppress tumor cell activity and remodel the immunosuppressive TME.

#### 4.1.3 Heterogeneity in extracellular vesicle origins influences ICD

##### 4.1.3.1 Tumor-derived extracellular vesicles modulate immune responses

The levels of extracellular vesicles in the body fluids of cancer patients are typically elevated. TEXs, secreted by cancer cells, are distributed throughout all body fluids, forming a communication network system (283). Extracellular vesicles originating from tumor cells primarily exhibit immunosuppressive properties. TEXs can carry CD39 and CD73, delivering them to target cells where they convert pro-inflammatory ATP into the immunosuppressive factor adenosine. CD39 Tregs exhibit upregulated immunosuppressive function due to increased adenosine production from TEX-delivered CD73 (284). TEXs in the TME also influence DC differentiation and maturation, converting them into negative regulators of immune responses. Tumor-derived extracellular vesicles carrying miR-203 downregulate TLR4 expression and TNF-α production in DCs, causing functional impairment (285). Multiple ligands carried by tumor extracellular vesicles interfere with NK cell functions such as recruitment and cytotoxic activity. For instance, AML extracellular vesicles transmit multiple inhibitory signals to NK-92 cells, significantly diminishing their migration toward tumors (286). Injection of extracellular vesicles derived from gastric cancer cell lines such as MKN-45 and MKN-28 increases the frequency of MDSCs and other cells while reducing the CD8^+^ T cell-to-NK cell ratio, establishing an immunosuppressive microenvironment in the lungs (287). TEXs may promote immune evasion; TEXs derived from the human cholangiocarcinoma cell line RBE can downregulate CD8^+^ T cell and NK cell numbers, inhibit TNF-α and perforin secretion, thereby weakening the antitumor activity of effector cells (288). However, tumor-derived extracellular vesicles can also activate the immune system to mediate tumor cell killing. Extracellular vesicles from plasma cell tumors expressing membrane-bound HSP70 can induce DC maturation, stimulate type 1 CD4^+^T and CD8^+^T cell responses, and induce NK cell cytotoxicity (289), thereby activating the immune system to kill tumor cells.

Tumor-derived extracellular vesicles predominantly exert inhibitory effects on immune cells, promoting the infiltration of immunosuppressive cells into the TME and thereby shaping an immune barrier and immune escape environment. However, extracellular vesicles carrying tumor antigens or DAMPs can also activate the immune response system, “heating up” the “cold” immune environment.

##### 4.1.3.2 The regulatory role of extracellular vesicles derived from immune cells in immunity

EVs derived from immune cells exert profoundly complex and ambivalent effects on tumor cells, playing a pivotal role in the TME. They can both transmit immune activation signals to stimulate antitumor immune responses that eliminate tumor cells and shape immunosuppressive environments that facilitate tumor cell immune escape and invasion. DCs process exogenous antigens within endosomal compartments, which subsequently fuse with the plasma membrane to form DC-derived EVs (DCexos) for secretion. DCexos can express functional peptide/major histocompatibility complex (MHC) class I and peptide/MHC class II complexes, thereby initiating specific T cell responses (290). DCexos carrying high levels of MHC class I molecules can induce antigen-specific CD8^+^ T cell responses, and EVs from monocyte-derived dendritic cells (MDDCs) exhibit enhanced T cell stimulatory capacity (290). DCexos promote NK cell proliferation and activation via IL-15Rα and NKG2D-dependent pathways, generating NK1.1^+^ cell-mediated antitumor effects (291).

NKcells are key effector cells of innate immunity, capable of secreting multiple antitumor cytokines such as IL-4, IFN-γ, FasL, and perforin to kill tumor cells (292). NK cells are frequently activated by EVs released from DCs. DC-derived EVs play a crucial role in NK cell activation: EVs from immature human DCs express NKG2D ligands (NKG2D-L), which bind to the NKG2D receptor on the surface of NK cells to activate them (291). Furthermore, TNF superfamily ligands (e.g., TNF, FasL) on DC-derived EVs can further activate NK cells and promote IFN-γ secretion (293). Concurrently, NK cell-derived EVs also exhibit antitumor activity. For example, EVs derived from NK-92MI cells (NK-92 Exo) express functional proteins such as perforin and FasL, secrete TNF-α, and exert cytotoxic effects on melanoma cells (294).

T cell-derived EVs can activate other immune cells, suppress immune responses, and participate in the licensing process of APCs (295). Engineered Jurkat T cells secreting IL-2-containing EVs exhibit significant changes in miR-181a-3p and miR-223-3p expression, which can reduce PD-L1 expression in melanoma and enhance CD8^+^ T cell immune sensitivity (296). CD4^+^ T cell-derived EVs carrying miR-25-3p, miR-155-5p, and other molecules can induce CD8^+^ T cell-mediated antitumor effects (297). However, T cell-derived EVs can also mediate immunosuppression, shaping a protumor microenvironment. CD8^+^ T cell-derived EVs can be internalized by APCs via pMHC-I/TCR interactions, thereby suppressing DC-mediated indirect CD8^+^ CTL responses (298). Treg-derived EVs downregulate M1 macrophage marker expression while promoting M2 marker expression (299). EVs from naive CD8^+^ CD25^+^ Tregs suppress DC-induced CTL responses and antitumor immunity in a B16 melanoma model (300).

**Figure 5:**
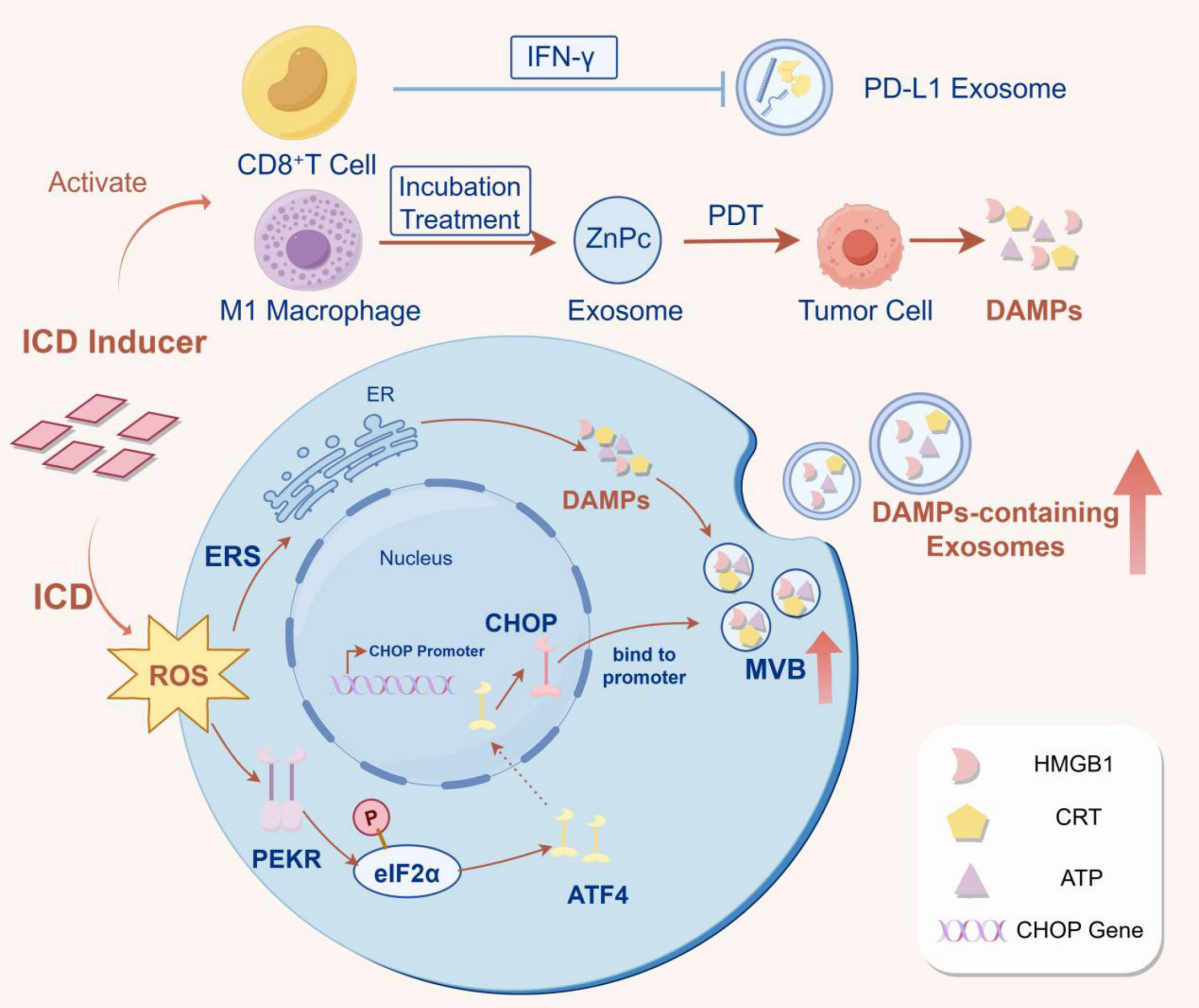
Effects of ICD on exosomes.

In tumor immunity, while certain immune cells exhibit duality under specific conditions, immune cells broadly fall into two categories: antitumor and protumor. As pivotal cells bridging innate and adaptive immunity, DCs frequently activate diverse immune cells such as NK cells and T cells for antitumor responses. EVs from antitumor immune cells are typically antitumor, while those from protumor immune cells exert the opposite effect. Engineering EVs from antitumor immune cells often enhances antitumor effects while preserving their original functions, such as by carrying ICD inducers. Conversely, inhibiting the secretion of EVs from protumor immune cells can alleviate the immunosuppressive TME. These two approaches offer novel strategies for improving clinical tumor therapy. (As illustrated in **Figure 5**).

Reading Pathway: ICD inducers activate ER stress and ROS-related pathways, inducing DAMPs that are released via exosomes, while simultaneously activating CD8^+^ T cells and M1 macrophages, thereby reinforcing ICD.

(This figure illustrates the molecular mechanisms underlying the reciprocal regulation between ICD and exosomes. Immunogenic cell death inducers trigger robust endoplasmic reticulum stress and elevated reactive oxygen species expression—two hallmark features of ICD—which collectively activate the PERK/eIF2α signaling pathway, thereby inducing DAMP generation and their release via exosomes derived from multivesicular bodies. Concurrently, ROS further activate the PERK/eIF2α/ATF4/CHOP pathway to induce ICD; within this pathway, PERK phosphorylates eukaryotic translation initiation factor 2α, promoting translation of activating transcription factor 4 and inducing expression of CCAAT/enhancer-binding protein homologous protein. DAM generated through this pathway can also be released via exosomes. Additionally, ICD inducers act directly on immune cells, promoting CD8^+^ T cell activation and interferon-γ secretion while suppressing exosomal PD-L1 release; they also enhance M1 macrophage activity. M1 macrophage-derived exosomes loaded with the photosensitizer zinc phthalocyanine can be employed for photodynamic therapy, inducing DAMP production in cancer cells and triggering ICD. Together, these mechanisms illustrate the synergistic interplay between ICD and exosomes in tumor immune regulation).

### 4.2 Extracellular vesicle-mediated ICD effects

Extracellular vesicle-mediated ICD can be induced under conditions such as radiotherapy, combination therapy, or novel therapeutic strategies. By releasing DAMPs and tumor antigens, it activates anti-tumor immune responses and reverses the immunosuppressive microenvironment. Extracellular vesicles loaded with REV and DOX, mimicking the nanoparticle EM@REV@DOX, promote dendritic cell maturation, regulate macrophage polarization, and activate T cells via ICD and cGAS-STING signaling pathways, offering novel insights for tumor immunotherapy (301). Additionally, a dual-delivery system based on bone marrow mesenchymal stem cell (BM-MSC) extracellular vesicles, internally loaded with galectin-9 siRNA and surface-modified with OXA prodrug, functions as an ICD inducer to reverse M2-like tumor-associated macrophage (M2-TAM)-mediated immunosuppression (302). Fusion of γδ-T cell extracellular vesicles with Ce6-loaded liposomes enables combination with PDT for targeted photoimmunotherapy. Under light irradiation, this system generates ROS within melanoma cells, inducing apoptosis and promoting ICD. This facilitates dendritic cell maturation and activates melanoma antigen-specific CD4^+^ and CD8^+^ cell responses, thereby enhancing antitumor immunity (303). Loading human neutrophil elastase (ELANE) and TLR3 agonist siltolide onto α-lactalbumin-engineered extracellular vesicles derived from breast cancer cells constructs the *in situ* DC vaccine HELA-Exos, which specifically induces ICD in breast cancer cells (127). Following HELA-Exos-induced ICD, tumor antigens and siltolide jointly activate *in situ* cDC1s, cross-presenting tumor antigens and triggering CD8^+^ T cell responses, demonstrating antitumor effects in triple-negative breast cancer (TNBC) patients (127). Furthermore, extracellular vesicles expressing HSPs exert immunostimulatory effects on NK cell function; for instance, HSP70^+^ extracellular vesicles derived from colon cancer promote NK cell migration and cytotoxicity (304).

Extracellular vesicle-mediated ICD effects are often achieved by engineering extracellular vesicles to carry ICD inducers or surface modifications that induce ICD, often in combination with other antitumor therapies to reverse resistance or enhance antitumor activity. Therefore, targeting extracellular vesicles to develop diverse extracellular vesicle-based antitumor therapies may represent a novel direction for future clinical treatments.

### 4.3 Extracellular vesicle-ICD-mediated intercellular communication network

#### 4.3.1 Tumor cell-immune cell crosstalk

##### 4.3.1.1 Tumor-derived EVs modulating DC function

TEXs exert dual regulatory effects on DC function, with specific outcomes dependent on the contents of extracellular vesicles, tumor microenvironment conditions, and DC subtypes. TEXs rich in tumor-associated antigens (TAAs) and neoantigens can deliver antigens to DCs, thereby stimulating antitumor immune responses. However, TEXs can also modulate the number and activity of Tregs and MDSCs within the TME, promoting immune suppression (305). Dendritic cell-derived extracellular vesicles expressing HCC antigens can induce immune responses and transform the TME into an immunostimulatory state, enhancing the cytotoxic activity of immune cells against tumor cells (306). Dendritic cell-derived extracellular vesicles containing MHC I and II, T-cell costimulatory molecules, and TAAs can activate CTL responses and inhibit tumor growth in mice (307). When dendritic cell-derived extracellular vesicles are activated via the TLR4 signaling pathway, they stimulate macrophages and dendritic cells, leading to enhanced antitumor immunity *in vivo* (308). TEXs can also shape immunosuppressive microenvironments, affecting DC activity and functional states to create favorable conditions for tumor growth. Microvesicles secreted by lung and breast cancer cell lines express PD-L1, which binds to PD-1 on DCs, inhibiting their phenotypic maturation and ability to migrate to lymph nodes (309). Similarly, melanoma-derived extracellular vesicles have been shown to reduce DC function and activity (310). Ovarian cancer extracellular vesicles can migrate to draining lymph nodes, be internalized by DCs, and subsequently suppress antigen-specific T cell proliferation (311). Furthermore, arginase-1 contained within extracellular vesicles can enhance DC arginine metabolism under IL-6 stimulation, thereby inhibiting T cell expansion and antigen presentation by depleting L-arginine (312).

Within the immune system, DCs occupy a central position as initiators and regulators of adaptive immune responses, serving as a bridge between innate and adaptive immunity. Tumor cell extracellular vesicles frequently contain bioactive substances such as TAAs and PD-L1, which modulate DCs by either promoting or suppressing their activity and function, thereby regulating immune system responses.

#### 4.3.2 Interactions between immune cells

##### 4.3.2.1 Macrophage polarization

Macrophages can be polarized into two distinct phenotypes based on their activation state: M1 and M2. The transition between these two states is a highly dynamic and reversible process. M1 macrophages exhibit pro-inflammatory properties and are activated by various stimuli to release large amounts of inflammatory mediators (313). Within the TME, TAMs predominantly adopt the M2 subtype, promoting tumor growth and metastasis by suppressing inflammatory responses (314). Nucleic acids derived from EVs play a crucial role in the M2 polarization of macrophages, facilitating the formation of an immunosuppressive microenvironment and tumor progression. Tumor-derived EV miR-21-5p suppresses RhoB expression and blocks the MAPK pathway, thereby inducing M2-like macrophage polarization, which correlates with poor prognosis in HCC patients (315). miR-660-5p carried by M2-type TAM-derived EVs promotes HCC tumorigenesis by regulating Krüppel-like factor 3 (KLF3), enhancing tumorigenic capacity in mouse models (316). In ovarian cancer (OC), EV-delivered miR-205 downregulates PTEN in macrophages, activates the PI3K/AKT/mTOR pathway, induces M2 polarization, and accelerates cancer cell invasion and metastasis (317). EV-derived miR-4669 promotes M2 polarization by upregulating sirtuin 1, thereby conferring sorafenib resistance to HCC cells, enhancing tumor invasiveness, and reinforcing the immunosuppressive microenvironment (318).

Tumor cell-derived EVs not only promote tumor proliferation, invasion, and metastasis but also regulate the polarization of macrophages toward the M2 phenotype within the TME, thereby accelerating tumor progression (319). The CCT6A protein in EVs derived from pancreatic ductal adenocarcinoma enhances the M2 phenotype of TAMs by activating the PI3K-AKT signaling pathway, which correlates with poor patient prognosis (320). PSM-E, which is highly expressed in serum and urine EVs from prostate cancer (PCa) patients, recruits RACK1 to inhibit FAK and ERK signaling pathways, thereby blocking macrophage M2 polarization and suppressing PCa proliferation, invasion, and metastasis (321). Targeting ASCT2 inhibits glutamine uptake, leading to intracellular ROS accumulation and subsequent activation of p38, Akt, and SAPK/JNK signaling pathways, which induces M1-like TAM polarization via EVs and promotes malignant progression in oral squamous cell carcinoma (OSCC) (322). β-elemene regulates SYNCRIP to promote miR-127-3p packaging into EVs, and EV-delivered miR-127-3p stimulates macrophage polarization toward the M1 phenotype by inhibiting ZC3H4 (323).

Tumor cells “educate” macrophages via EVs, transforming them into protumor M2 cells. Intervening in this process or delivering “re-education” signals via EVs holds promise for reshaping the tumor immune microenvironment and improving clinical outcomes. Targeting tumor cell EV production and release, or utilizing engineered EVs to deliver pro-M1 signals, represents a viable therapeutic strategy.

##### 4.3.2.2 NK cell activation

NK cells, as key components of innate immunity, can be rapidly activated without specific immune recognition to directly kill tumor cells and virus-infected cells. They also regulate immune responses by secreting cytokines, making them crucial effector cells in the body’s defense against tumors and infections. EVs, as vital mediators of intercellular communication, significantly regulate NK cell functional states. Tumor-derived EVs frequently suppress NK cell activity. Studies indicate that mouse NK cells exhibit markedly reduced killing capacity after co-incubation with tumor-derived EVs (324). In human peripheral blood mononuclear cell (PBMC)-derived NK cells and the NK-92 cell line, EVs from clear cell renal cell carcinoma and pancreatic cancer activate the TGF-β-Smad2/3 signaling pathway via TGF-β receptors, inducing Smad2/3 phosphorylation and thereby suppressing NK cell function (325, 326). However, engineered EVs such as eNK-EXO not only deliver cisplatin to enhance toxicity against drug-resistant ovarian cancer cells but also reverse NK cell suppression in the TME (327). Conversely, NK cell-derived EVs (NKEVs/NK-EVs) exhibit multifaceted immunomodulatory and antitumor effects. In severe combined immunodeficiency (SCID) mouse models, NKEVs significantly suppressed lymphoma proliferation and induced tumor cell apoptosis (328). EVs expressing receptors such as CD56 and NKG2D can “educate” NK cells, enhancing their cytotoxicity against neuroblastoma (329). Furthermore, NK-EVs promote CD4^+^ T cell polarization toward the Th1 phenotype and enhance the antigen presentation and co-stimulatory capacity of DCs (330). DC-derived EVs (Dex/Dcex) also participate in NK cell activation. Dcex can directly induce tumor cell apoptosis through surface-expressed molecules such as TNF and FasL, while simultaneously activating NK cells (293). Meanwhile, DCs promote NK cell proliferation and IFN-γ secretion via IL-15Rα, and directly bind to and activate NK cells through NKG2D ligands to exert antitumor metastasis effects (291).

**Figure 6:**
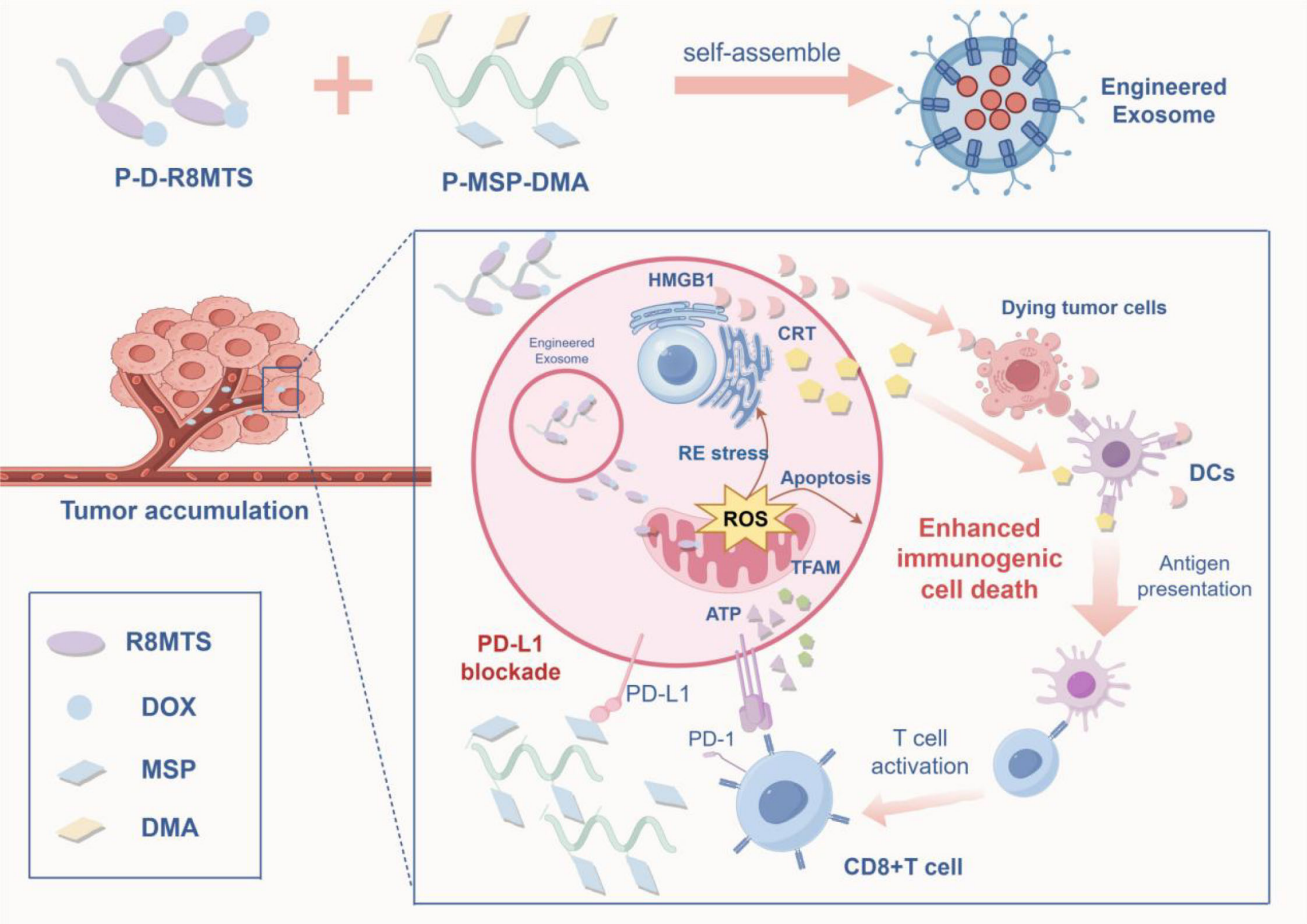
Effects of exosomes on ICD.

As a key component of innate immunity, NK cells can function as recipient cells to receive EVs from tumor cells or DCs, or as donor cells to supply EVs to T cells and other immune cells, thereby regulating antitumor immunity. A major advantage of NK cells is their ability to kill tumor cells without requiring specific immune recognition for activation. (As illustrated in **Figure 6**).

Reading Pathway: Engineered exosomes (assembled from P-D-R8MTS and P-MSP-DMA) block PD-1/PD-L1 interactions and activate CD8^+^ T cells. Simultaneously, P-D-R8MTS induces targeted ROS generation, triggering ICD and activating antitumor immunity.

(This figure illustrates the mechanism by which engineered exosomes regulate immunogenic cell death. Mitochondria-targeting heteropeptide-modified N-(2-hydroxypropyl) methacrylamide copolymer-doxorubicin conjugates and anionic PD-L1-blocking copolymers assemble to form engineered exosomes. P-D-R8MTS stimulates PD-L1 upregulation on tumor cells, thereby inhibiting CD8^+^ T cell cytotoxicity. However, within the acidic tumor microenvironment, dimethylacrylamide dissociates from P-MSP-DMA, allowing P-MSP to block the PD-1/PD-L1 pathway. This reverses the PD-L1 upregulation induced by P-D-R8MTS, alleviates tumor immunosuppression, and restores CD8^+^ T cell cytotoxic activity. After entering tumor cells, engineered exosomes release P-D-R8MTS, which targets mitochondria to induce ROS production, triggering mitochondrial dysfunction and initiating ICD. Concurrently, damaged mitochondria release mitochondrial damage-associated molecules, enhancing immune responses and ICD efficacy. In addition, ROS induce endoplasmic reticulum stress, promoting DAMP release, enhancing APC phagocytosis, and potentiating T cell cytotoxicity. After tumor cell apoptosis, tumor-derived exosomes carrying antigens are presented by DCs, activating CTLs and inducing antitumor immunity. Collectively, this figure illustrates the mechanism by which engineered exosomes coordinately regulate ICD through multiple pathways to remodel antitumor immune responses).

### 4.4 Translational framework for extracellular vesicles and ICD interaction

#### 4.4.1 Challenges and obstacles

The synergistic interaction between extracellular vesicles and ICD holds significant implications for clinical tumor immunotherapy, yet its current application faces numerous challenges. Although ICD activates the immune system and its released DAMPs exhibit strong immunogenicity, the heterogeneous origins of extracellular vesicles enable tumor cells and immunosuppressive cells to release DAMPs like HMGB1 via extracellular vesicles. These DAMPs can function as tumor self-rescue signals, promoting metastasis. Furthermore, extracellular vesicle contents such as miRNAs can transmit pro-tumor and drug-resistant effects (254, 262). The immunosuppressive TME within the body is highly complex. While ICD activates anti-tumor immunity, extracellular vesicles originating from tumor cells and immunosuppressive cells within the TME counteract its effects. These vesicles can even “educate” other immune cells to undergo functional conversion toward tumor promotion, such as TAMs polarizing into M2 macrophages (317). Furthermore, naturally occurring extracellular vesicles exhibit limited specificity in targeting. The large-scale, stable production of high-quality engineered extracellular vesicles remains technically challenging. Simultaneously, precisely controlling the targeted release of ICD inducers loaded within extracellular vesicles currently presents significant difficulties.

#### 4.4.2 Clinical translation and future directions

Currently, engineered extracellular vesicles represent the most promising strategy for combination therapies involving extracellular vesicles and ICD. Through bioengineering, the surface molecules of extracellular vesicles can be tailored to enhance targeting specificity, or the vesicles can be loaded with ICD-related immunostimulatory agents to reverse or alleviate immunosuppressive barriers within the TME. As mentioned above, HELA-Exos can act as effective drug delivery vehicles that induce ICD in cancer cells, enhance the release of DAMPs and tumor-associated antigens, and activate local DCs in co-culture systems consisting of breast cancer patient PBMCs and autologous tumor organoids (127). Engineered extracellular vesicles further address the common clinical challenge of apoptosis resistance in antitumor therapy. A hybrid extracellular vesicle delivery system (IHEL) combining hemin and the phototherapeutic agent IR780 induces ICD and promotes M1 macrophage polarization. Under IR780-mediated phototherapy, this system shifts the predominant apoptotic anticancer mechanism to a combined ferroptosis-apoptosis mode, thereby helping to overcome apoptosis resistance (331), while providing both theoretical basis and practical strategy for photodynamic anticancer therapy. A bioengineered extracellular vesicle drug delivery nanoplatform (Apatinib-Exo-aPD-L1) has been shown to improve therapeutic outcomes in CRC by triggering ICD and promoting DC maturation, thus increasing the infiltration and activation of CTLs within the TME. Concurrently, this platform suppresses the abundance and function of Tregs and MDSCs, effectively converting “cold” immunosuppressive TME into “hot” immunogenic TME (332). Future strategic directions will likely focus on rational combination therapies: using extracellular vesicles loaded with ICD inducers to activate antitumor immunity while simultaneously blocking extracellular vesicle-mediated immunosuppressive signaling. Targeting tumor cells or immunosuppressive cells to reduce their extracellular vesicle secretion or remodel their cargo to function as “endogenous tumor vaccines” also represents a feasible approach. We propose that future therapeutic strategies should center on two core principles: first, blocking the tumor-promoting effects of extracellular vesicles to attenuate therapeutic resistance within the TME; second, utilizing engineered extracellular vesicles to actively “build” effective antitumor immune responses, thereby remodeling immunosuppressive TME and restoring robust antitumor immune reactivity. (As illustrated in **Table 6**).

**Table 6:** Drug/intervention strategies targeting extracellular vesicle-induced cytoplasmic death-related mutual regulation.

Strategies/agents	Mechanism of action	Research model	Related nucleic acids/pathways/cells	Results and outcomes	References
5-Aminolevulinic acid photodynamic therapy (ALA-PDT)	Regulates miR-34a-mediated HMGB1 exosome secretion	HPV-positive cervical cancer cells	miR-34a/HMGB1	Inhibits proliferation and promotes apoptosis of HPV-positive cervical cancer cells	(257)
HA nanoparticles	Nanoparticles loaded with miRNA-125b regulate macrophage polarization	Mouse model of non-small cell lung cancer	M1 Macrophages	Promote M1 polarization and enhance antitumor efficacy	(265)
CD44/EGFR dual-targeting (HA) nanoparticles (NPs)	Delivers plasmid DNA to increase wt-p53 and miR-125b gene expression	Human lung adenocarcinoma cell model	miRNA-125b/wtp53/macrophages	Induces M1 polarization and inhibits tumor growth	(266)
Endosomal deubiquitinase USP8	Endosomal deubiquitinase USP8 interacts with CTLA-4	Mouse tumor model	CD4^+^ T cells	Lifting T-cell activation inhibition	(274)
Tamoxifen	Upregulates miR-329 expression in cancer cell exosomes, inhibiting histone demethylase KDM1A	MCF-7 breast cancer cell model	miR-329/KDM1A	Reduces angiogenesis and exerts antitumor effects	(276)
Cell-free DNA fragments from certain tumors	Methylation status and structural features of cfDNA fragments reflect chromatin activity in cancer cells	Cancer cell models	cfDNA	Decoding cfDNA epigenetic features holds promise for early cancer detectionn	(282)
Extracellular vesicles from plasmacytoma cells expressing membrane-bound HSP70	Extracellular vesicles from plasmacytoma cells expressing membrane-bound HSP70	Plasmacytoma model	DCs/CD4^+^ T cells/CD8^+^ T cells	Activates tumor-killing effects of the immune system	(289)
NK-92MI cell-derived extracellular vesicles (NK-92 Exo)	NK-92 Exo can express functional proteins such as perforin and FasL, and secrete TNF-α.	Melanoma model	Perforin/FasL/TNF-α	Exert cytotoxic effects on melanoma cells	(294)
Engineered Jurkat T cells	Altered expression of miR-181a-3p and miR-223-3p reduces PD-L1 expression in melanoma.	Melanoma model	miR-181a-3p/miR-223-3p	Enhances CD8^+^ T cell immune sensitivity	(296)
EM@REV@DOX (extracellular vesicles loaded with REV and DOX)	Promotes DC maturation, regulates macrophage polarization, and activates T cells		ICD/cGAS-STING	Provides new insights for tumor immunotherapy	(301)
BM-MSC extracellular vesicle delivery system (ICD inducer)	Loaded with galectin-9 siRNA and modified with oxaliplatin (OXA) prodrug	M2 macrophage model	M2-like tumor-associated macrophages	Reverses M2-like Tumor-Associated Macrophage-Mediated Immunosuppression	(302)
γδ-T cell extracellular vesicles fused with Ce6-loaded liposome membranes combined with PDT	Induces ICD, promotes DC maturation, and activates CD4^+^ and CD8^+^ T cell responses.	Melanoma cell model	ICD/multiple immune cells	Enhances antitumor immunity	(303)
*In situ* DC vaccine HELA-Exos (ELANE and hilrtol)	Induces ICD in breast cancer cells	Breast cancer cell model	ICD	Induces ICD in breast cancer cells	(127)
Dendritic cell-derived extracellular vesicles	Exosomes transport active molecules and activate immune cells	Tumor cells and mouse tumor models	Hepatocellular carcinoma (HCC) antigens and other bioactive substances interact with multiple immune cells	Alleviates immunosuppressive TME; activates antitumor immunity	(306–308)
Extracellular vesicle PSM-E	Recruits RACK1 and inhibits FAK and ERK	Clinical patients	RACK1/FAK/ERK	Inhibits PCa proliferation and invasion	(321)
β-Elemene	Regulates SYNCRIP to promote packaging of miR-127-3p into exosomes	Non-small cell lung cancer model	SYNCRIP/miR-127-3p/M1Macrophages	miR-127-3p inhibits ZC3H4, stimulating macrophage polarization toward M1 phenotype	(323)
Engineered extracellular vesicles eNK-EXO	Delivers cisplatin to enhance toxicity against drug-resistant ovarian cancer cells	Ovarian cancer cell model	Natural Killer cells	Induces tumor cell apoptosis	(327)
NK cell-derived extracellular vesicles (NKEV)	NKEV Significantly inhibits lymphoma proliferation	SCID mouse model	—	Induces tumor cell apoptosis	(328)
NKEV	Promotes CD4^+^ T cell polarization toward Th1 phenotype and enhances DC function	Mouse tumor model	CD4^+^T/DCs cells	Enhances immune cell activity	(330)
Dendritic cell-derived extracellular vesicles	Induce tumor cell apoptosis and activate NK cells	Clinical trial models, etc.	TNF/FasL/IFN-γ, etc.	Exerts pro-apoptotic and anti-metastatic effects	(291, 293)
IHEL (hemin/IR780 phototherapy agent)	Triggers ICD to elicit anticancer immunity; increases M1 macrophage polarization	Melanoma model	ICD	Shifts anticancer paradigm from apoptosis-dominant to ferroptosis/apoptosis combined mode	(331)
Apatinib-Exo aPD-L1	Triggers ICD and activates multiple immune cells	Mouse model of colorectal cancer	ICD/Multiple Immune Cells	Ameliorates TME immunosuppression	(332)

## 5 Challenges and obstacles

Currently, research in this field still faces numerous challenges and obstacles. “Hot tumors” are characterized by abundant infiltration of immune effector cells and an immune-activated state (333). However, the immunosuppressive nature of the TME—marked by the enrichment of MDSCs and Tregs, along with microenvironmental factors such as hypoxia and acidosis—continuously impairs immune cell function, making the conversion from “cold” to “hot” tumors challenging to achieve (334). Furthermore, the “dual physical-immunological barrier” constructed by stromal cells within the TME represents a core reason why certain tumors resist the transition from “immune desert” to “hot tumor.” Only by targeting the stromal barrier—for instance, by degrading physical obstacles and reversing immunosuppressive states—can the path be cleared for the infiltration and activation of immune effector cells, thereby laying a foundation for the establishment of a “hot tumor” to support subsequent immunotherapy (8).

Exosomes also exhibit functional duality—tumor-derived exosomes carry immunosuppressive molecules such as PD-L1 and TGF-β that inhibit immune responses (335–337). Currently, effective strategies for selectively harnessing or blocking specific exosome subpopulations remain lacking. Furthermore, as critical mediators of intercellular communication within the TME, exosomes possess intrinsic targeted delivery potential that offers unique opportunities for precision cancer therapy. However, achieving precise targeting within the TME remains a significant challenge. As Li et al. pointed out, although certain tumor-derived exosomes can be preferentially taken up by homologous or specific cell types, this natural targeting capacity is generally inefficient and non-specific, failing to meet the requirements for precision therapy and thus constituting a major obstacle to clinical translation (338). To overcome the limitations of native exosomes in therapeutic applications, it is necessary to construct “engineered exosomes” that can efficiently load therapeutic small molecules, nucleic acids, or proteins and endow them with specific targeting ligands (339). This perspective is echoed by Alptekin et al. in their in-depth discussion on the application of engineered exosomes in tumor immunology research, where they elaborated on various engineering strategies to precisely regulate exosome targeting specificity, enabling active targeting of specific cell types or structures within the TME (340). Although engineered exosomes offer significant advantages in targeting specificity and drug loading efficiency, they still face bottlenecks including large-scale production, standardization of drug loading, and unclear *in vivo* kinetics. Therefore, future research should continue to explore their characterization, mechanisms of action, and therapeutic potential (341, 342).

Furthermore, the induction of ICD exhibits significant uncontrollability. Different tumors display heterogeneous responses to ICD inducers such as chemotherapy, radiotherapy, or photodynamic therapy, and key signals released during ICD—including CRT and HMGB1—may be degraded by enzymes within the TME. Moreover, potent ICD inducers must balance immunogenicity against the risk of systemic inflammatory responses or damage to normal tissues (343, 344). Concurrently, the protumor functions of exosomes may counteract the immune-activating effects of ICD. On one hand, exosomal PD-L1 mediates T cell suppression. Exosomes secreted by tumor cells and TAMs carry PD-L1, which directly inhibits T cell activation and cytotoxicity by engaging the PD-1 receptor on the surface of T cells (345). Further studies have revealed that tumor cells and TAMs reciprocally transfer PD-L1 via exosomes, establishing a positive feedback loop that continuously amplifies the immunosuppressive microenvironment (346). On the other hand, NK cell function is also suppressed. Leukemia-derived exosomes inhibit NK cell cytotoxicity through TGF-β signaling: exosomal TGF-β downregulates the expression of activating receptors on NK cells and reduces granzyme B secretion (347). Some scholars have emphasized that tumor exosomes can also block NK cell activation and impair their tumor surveillance function by carrying other immunosuppressive molecules (348). Furthermore, tumor exosomes promote immune evasion through multiple additional pathways—including inducing Treg expansion and MDSC activation (334), delivering immunosuppressive factors such as TGF-β and IL-10, driving M2 macrophage polarization (349), and suppressing DC maturation—all of which act synergistically to inhibit antitumor immune responses and accelerate disease progression (350). This further increases the complexity of therapeutic design.

In terms of mechanistic research, future efforts should integrate multi-omics technologies—including genomics, transcriptomics, proteomics, and metabolomics—to comprehensively decipher the complex molecular regulatory network underlying the “TME-exosome-ICD” axis. For instance, the integration of multidimensional biomarkers could guide precision patient stratification and personalized treatment (351). Concurrently, spatiotemporal dynamic analysis techniques can be employed to investigate the interactions among these three components across different temporal and spatial dimensions, providing deeper insights into their dynamic evolution at various stages and sites of tumor progression, thereby establishing a more robust theoretical foundation for optimizing therapeutic strategies. Regarding therapeutic development, several key technical bottlenecks remain to be overcome. Critical challenges include: how to efficiently and specifically induce ICD in tumor cells while minimizing damage to normal tissues; how to achieve large-scale, high-purity production of therapeutically active exosomes with precise *in vivo* delivery; and how to overcome immunosuppressive factors within the TME to ensure that ICD-induced and exosome-mediated immune activation signals are effectively transmitted and executed. Future research should focus on the development of intelligent delivery systems, innovation in dynamic monitoring technologies, and integration across multiple scales of mechanism. Only through such comprehensive approaches can the efficient synergistic breakthrough of this “iron triangle” be achieved.

## 6 Summary and outlook

### 6.1 The innovation of the “Iron Triangle” strategy

While single-agent intervention strategies targeting the TME, exosomes, or ICD have all been reported in preclinical studies, the “iron triangle” synergistic strategy proposed in this review represents a fundamental departure from these conventional unimodal approaches, with its core innovation lying in the paradigm shift from “single-node intervention” to “coordinated network regulation”.

Significant differences in gene expression, metabolic status, and immunogenicity exist not only across distinct regions within a tumor but also among individual tumor cells (352). This intratumoral heterogeneity underscores the inherent limitation of single-agent strategies targeting a specific component or signaling pathway within the TME: such approaches often only transiently suppress a subset of tumor cells, while resistant populations survive and proliferate, ultimately driving therapeutic resistance and recurrence (353). For instance, although targeting TAMs may potentiate antitumor immunity, the presence of other immunosuppressive cells or mechanisms within the TME can limit its efficacy (354). Similarly, while conventional immune checkpoint inhibitors can reinvigorate exhausted T cells, clinical studies indicate that defects in the antigen presentation machinery of tumor cells render T cells unable to effectively recognize malignant cells even when the PD-1/PD-L1 axis is blocked, leading to therapeutic resistance Similarly, while conventional immune checkpoint inhibitors can reinvigorate exhausted T cells, clinical studies indicate that defects in the antigen presentation machinery of tumor cells render T cells unable to effectively recognize malignant cells even when the PD-1/PD-L1 axis is blocked, leading to therapeutic resistance (355). ICD inducers alone can release DAMPs to activate immunity; however, these critical signals are highly susceptible to neutralization by metabolites within the TME, thereby attenuating the immunogenicity of ICD (356). Furthermore, strategies targeting exosomes in isolation attempt to block “bad signals” but fail to actively establish “good signals” (357). Notably, TME-derived exosomes tend to be immunosuppressive, whereas ICD-induced exosomes may be immune-activating (358). These two classes of exosomes coexist within the body, exerting antagonistic functions, and their net effect remains highly unpredictable.

The innovation of the “iron triangle” strategy proposed in this review lies in its construction of a positive feedback loop: ICD initiates antitumor immunity, exosomes efficiently disseminate activating signals throughout the TME, and the engineered or remodeled TME in turn better supports immune cell infiltration and sustained effector function. This networked regulatory effect far exceeds the simple sum of the individual contributions of the three components, constituting a mechanistic shift from “linear suppression” to “network amplification”. Moreover, this review emphasizes that by intervening in the TME, the cargo composition of exosomes can be remodeled—transforming them from carriers of suppressive signals to vehicles enriched with activating signals. This repurposes native tumor-derived exosomes into antitumor messengers, achieving a qualitative leap from “functional antagonism” to “synergistic conversion”.

In summary, the synergistic strategy integrating TME, exosomes, and ICD does not represent a simple additive combination of the three components. Rather, by elucidating their interconnected network of interactions and leveraging the bridging function of exosomes, this approach achieves an organic integration of TME remodeling with ICD induction. This constitutes a paradigm shift from “isolated confrontations” to “systemic regulation,” offering a more promising framework for overcoming immunotherapy resistance.

### 6.2 Challenges and obstacles

In light of the multiple challenges that persist—such as the dynamic heterogeneity of the TME, the functional duality of exosomes, and the fact that ICD-induced immunogenicity is jointly constrained by the metabolic environment of the TME and exosomal signals—future research should focus on the following directions:1)Deciphering the dynamic regulatory network of the “iron triangle.” By integrating technologies such as single-cell sequencing and spatial transcriptomics, the spatiotemporal correlations among TME metabolites, exosomes, and ICD markers across different tumor subtypes should be elucidated. Identifying the regulatory hierarchy of key nodal molecules will provide a theoretical foundation for multi-target combination strategies. 2)Developing intelligent targeted delivery systems. Leveraging the natural targeting capacity of exosomes, engineered exosomes responsive to TME characteristics should be designed to achieve co-delivery of ICD inducers and immunomodulators, while simultaneously blocking exosome-mediated immunosuppressive signals. 3)Advancing the clinical translation of combination therapies. Synergistic strategies integrating “TME remodeling—exosome regulation—ICD activation” should be explored. For example, anti-angiogenic therapy could improve vascular normalization to enhance exosome delivery efficiency; combining this with photothermal therapy could induce ICD and activate the cGAS-STING pathway, while adjunctive use of exosome-derived DC vaccines could amplify antigen presentation effects. Ultimately, this approach aims to achieve “hot tumor” conversion and the establishment of immunological memory.

The “iron triangle” composed of TME, exosomes, and ICD represents a potent synergistic strategy for cancer therapy. By harnessing ICD to initiate robust tumor-specific immune responses, utilizing engineered exosomes as precision delivery tools and immunomodulators, and targeting interventions to remodel the immunosuppressive TME, these three elements form a positive feedback loop that collectively overcomes the barriers of “cold tumors.” This strategy holds promise for significantly enhancing both the quantity and quality of T cell infiltration, transforming “immune deserts” into “hot tumors,” thereby greatly potentiating the efficacy of cancer immunotherapy and offering new hope for conquering solid tumors.

**Figure 7:**
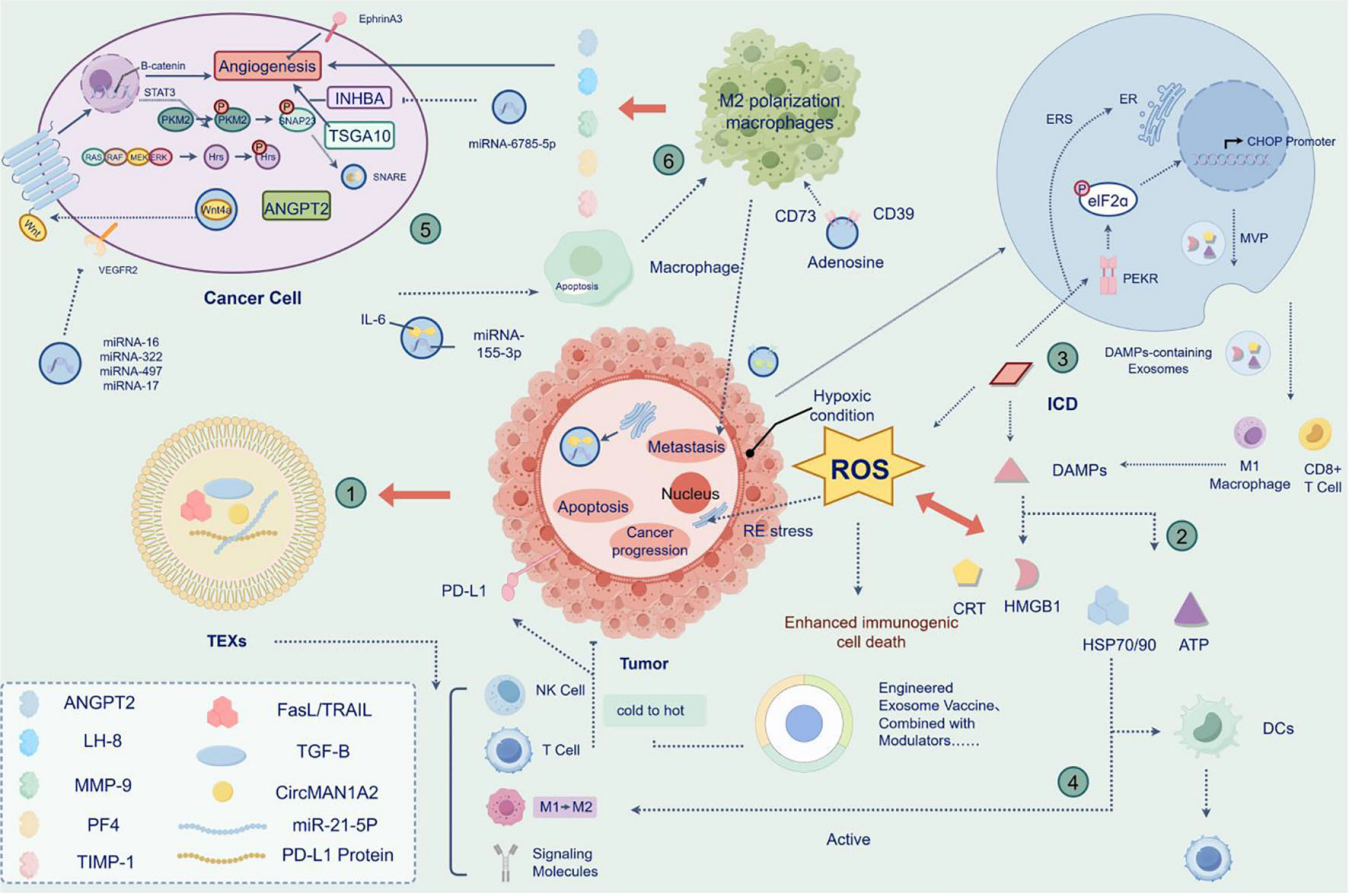
ROS-regulated TME-exosome-ICD interplay network.

With deeper insights into exosome engineering technologies and ICD induction mechanisms, this “iron triangle” synergistic strategy is poised for broader development prospects. In future clinical practice, precise modulation of ICD induction and exosome function may enable the development of novel cancer immunotherapies, improving therapeutic efficacy and patient survival rates, and ultimately realizing precision oncology. (As illustrated in **Figure 7**).

Reading Pathway:Starting from central tumor cells and the core regulatory axis of ROS under hypoxic conditions, core events such as ER stress and ICD triggered by ROS lead to the molecular mechanisms of TEX-mediated immunosuppressive microenvironment formation, ICD-mediated “cold-to-hot” tumor conversion, ERS/ROS pathway-enhanced ICD, engineered exosome-activated antitumor immunity, oncogenic signal-induced angiogenesis, and hypoxia-driven macrophage M2 polarization and tumor metastasis, following the sequence numbered ① to ⑥.

(Hypoxic conditions within the TME induce tumor cells to generate abundant ROS, which serve as a central hub of the entire regulatory network. ROS directly trigger ER stress and ICD, mediate core biological processes including tumor progression, metastasis, and apoptosis, and simultaneously regulate interactions between tumor and immune cells. ROS drive tumor cells to release TEXs carrying signaling molecules such as PD-L1, TGF-β, and miR-21-5p, which directly suppress the antitumor activity of NK cells and T cells while inducing macrophage polarization toward mixed M1/M2 phenotypes, thereby establishing a locally immunosuppressive TME.

ROS induce ICD in tumor cells, releasing DAMPs including CRT, HMGB1, ATP, and HSP70/90, which recruit and activate M1 macrophages and CD8^+^ T cells, converting immunosuppressive “cold tumors” into immune-activated “hot tumors” and initiating antitumor immune responses. ROS-triggered ER stress activates the CHOP promoter via the PERK–eIF2α signaling pathway, further promoting tumor cell release of DAMP-containing exosomes and establishing a positive feedback loop that continuously amplifies the immune-activating effects of ICD.

Based on exosome regulatory mechanisms within the TME, targeted intervention using engineered exosome vaccines or modulators can directly activate the antitumor activity of DCs and downstream T cells and NK cells, achieving artificially regulated immune activation. Meanwhile, oncogenic signaling pathways within tumor cells, under ROS regulation, upregulate angiogenic factors such as ANGPT2 via the INHBA–TSGA10 axis while releasing molecules including miRNA-6785-5p, ultimately triggering tumor angiogenesis to provide nutritional support for tumor proliferation and metastasis.

Under hypoxic/ROS conditions, tumor cells release factors including IL-6 and miR-155-3p, inducing macrophage polarization toward the M2 phenotype through the CD39/CD73–adenosine pathway. M2 macrophages in turn promote tumor metastasis via signaling molecules such as EphrinA3, forming a reciprocal regulatory loop between tumor cells and macrophages that accelerates tumor progression).

### 6.3 Document search

1. Literature sources: PubMed (biomedical literature database), Web of Science (SCIE, Science Citation Index Expanded), Chinese Biomedical Literature Database (CBM), China National Knowledge Infrastructure (CNKI); the search period focused on January 1, 2010 to March 1, 2026. The language was limited to Chinese and English literature to ensure data homogeneity.

2. To comprehensively retrieve relevant literature, a cross-search strategy based on keywords was employed. Chinese search terms were set as follows: (exosomes) AND (immunogenic cell death OR immune cells OR immunotherapy) AND (tumor microenvironment). English search terms included: exosomes; (immunogenic cell death OR ICD OR immune cells OR T cells OR B cells OR neutrophils OR macrophages); tumor microenvironment; cold tumor; hot tumor; immune desert, etc. The search strategy combined subject headings (e.g., MeSH terms) and free terms, and Boolean operators (AND/OR/NOT) were applied to construct precise search equations. The search syntax was adjusted according to the characteristics of different databases, such as truncation in PubMed and synonym expansion in CNKI.

3. The initial search yielded a total of 9763 relevant literature citations. Two researchers independently performed a preliminary screening of the titles and abstracts of all retrieved documents, and then read the full text of the documents screened in the first round to conduct a second screening based on the inclusion criteria, while recording the reasons for rejection. Duplicate citations were removed: all retrieved citations were imported into NoteExpress software for duplicate checking, and 7127 were removed after deduplication. 8631 were excluded in the first round of screening, and 1132 were retained in the first round of screening.

(Preliminary screening: According to the exclusion criteria and inclusion criteria, the titles and abstracts of the retrieved entries are initially read to eliminate ineligible literature such as conference papers, technical reports, evaluation studies, and diseases that do not match).

Secondary screening: According to the exclusion and inclusion criteria, the full text of the obtained literature was carefully read, and 754 articles that did not meet the inclusion criteria were excluded, and finally 378 articles that met the inclusion criteria were obtained. Inclusion criteria: The research content focuses on the cross-regulatory mechanism of exosomes and immunogenic cell death in the tumor microenvironment. The types of research include: basic experiments (cell or animal models) to reveal the molecular pathways of their interaction; clinical studies (such as cohort studies, RCTs) to explore the efficacy of combination therapy (such as exosomes+ICD inducers); and mechanistic studies to analyze the effects of cross-regulatory mechanism of exosomes and immunogenic cell death on the tumor microenvironment (such as T cell infiltration and DC Maturation). The types of literature are original research papers (Articles), reviews, meta-analyses, clinical studies, mechanistic studies, etc., and conference abstracts, reviews, and case reports are excluded. Exclusion criteria: The research subjects are non-tumor diseases (such as neurodegenerative diseases); the research content is not related to the tumor microenvironment; the data is incomplete or there are significant methodological deficiencies (such as insufficient sample size and no statistical analysis). Then, using specific data collection forms, the researchers extracted relevant data from each study, including basic information about the article (first author, year of publication, journal name, and type of study); the experimental model of the research design (e.g., cellular animal model or patient), interventions (e.g., Oxaliplatin-induced immunogenic cell death, photodynamic therapy); key regulators of the core mechanism [e.g., DAMPs (CRT, HMGB1, ATP, HSPs), miRNA(miR-21, miR-127-3p), immune checkpoint molecules (PD-L1, CTLA-4), signal pathways (e.g., cGAS-STING, PI3K-AKT, TGF-β/Smad2/3), immunosuppressive cells (Tregs, MDSCs, TAMs)]; and the main findings of immunogenic cell death’s promotion/inhibition of immune cell changes, remodeling effect on the tumor microenvironment, and synergistic effect of combination therapy, as well as the limitations of the experimental model (e.g., lack of clinical verification) and unresolved scientific questions.

Meta-analysis results showed that exosomes carry key factors such as DAMPs, miRNAs and immune regulatory molecules, and achieve bidirectional effects of positive immune activation and negative immune suppression through core signaling pathways such as cGAS-STING and NF-κB signal.
